# Reasons for Non-Enrollment in Treatment among Multi-Drug Resistant Tuberculosis Patients in Hunan Province, China

**DOI:** 10.1371/journal.pone.0170718

**Published:** 2017-01-23

**Authors:** Zuhui Xu, Tao Xiao, Yanhong Li, Kunyun Yang, Yi Tang, Liqiong Bai

**Affiliations:** 1 Department of tuberculosis control, Tuberculosis Control Institute of Hunan Province, Changsha city, Hunan province, China; 2 Department of MDR-TB internal medicine, Hunan Chest hospital, Changsha city, Hunan province, China; 3 Department of director’s office, Tuberculosis Control Institute of Hunan Province, Changsha city, Hunan province, China; Chinese Academy of Medical Sciences and Peking Union Medical College, CHINA

## Abstract

In 2015, only 49% of notified multi-drug resistant tuberculosis (MDR-TB) patients in China were estimated to have initiated treatment, compared with 90% of those worldwide. A case-control study was conducted to identify the reasons for non-enrollment in treatment among MDR-TB patients in Hunan province, China. All detected MDR-TB patients registered in designated MDR-TB hospitals in Hunan province from 2011 to 2014 were included and followed until June 2015 to determine their treatment status. Approximately 33.8% (482/1425) of patients were not enrolled in standardized treatment. Factors associated with lower enrollment rate were: age greater than 60 years, living in rural area, unemployed or occupation unreported. Of those who were not enrolled in MDR-TB treatment, the primary reasons for non-enrollment included economic hardship (23.0%), out-migration for work (18.0%), concerns about work and studies (13.7%), and the belief that they were cured after undergoing drug-sensitive TB treatment (12.4%). Therefore, comprehensive strategies targeting priority populations, especially those enhancing treatment affordability and availability, need to be implemented to improve MDR-TB control.

## Introduction

Multi-drug resistant tuberculosis (MDR-TB) is one of the greatest challenges to controlling tuberculosis worldwide [1]. China has a serious epidemic of drug-resistant tuberculosis [2]. According to the 2015 Global Tuberculosis Report [1], only 49% of notified MDR-TB cases in China were enrolled in MDR-TB treatment. In Hunan Province, free treatment has been provided to MDR-TB patients since the second half of 2011 with funding from the New Rural Cooperative Medical System (NRCMS) and the Global Fund Tuberculosis Program. However, the rate of enrollment among notified cases remains low. Accordingly, this study followed all identified and notified MDR-TB patients in Hunan Province for approximately four years to identify the reasons for non-enrollment in MDR-TB treatment.

## Methods

### Study population

All MDR-TB patients registered between 2011 and 2014 in designated MDR-TB specialist hospitals (the majority of whom were registered in one provincial tuberculosis hospital) were enrolled as the study population. The methods used for MDR-TB detection included phenotypic drug susceptibility testing (based on solid culture and liquid culture techniques) and molecular methods (line probe assay). Mycobacterium tuberculosis (MTB) strains that were found to be resistant to both isoniazid (INH) and rifampicin (RMP) using any of the aforementioned methods were considered MDR-TB.

### Data sources

Four data sources from the designated MDR-TB hospitals were used to extract patient information: the “Drug Susceptibility Testing (DST) Results Registry form”, which documents all DST results, including diagnoses of MDR-TB; the “MDR-TB Case Registry form”, which includes data on the treatment enrollment of MDR-TB cases; the self-designed "MDR-TB Patient Tracing Registry form of Hunan Province", which includes information on the reasons for non-enrollment in treatment; and the Chinese Internet-based TB Management Information System, which includes data on patient sex, age, occupation, residence, and treatment history. For the analyses, age groups were categorized as ≤20, 21–40, 41–60 and ≥61 years. Each patient’s current location was categorized as central, northern, southern, southwestern, western, other province and unknown based on their socioeconomic status within the province and the travel distance required for MDR-TB care. The four forms were linked by CDC staffs using patient “patient name”, “sex”, “age”, and “living address “to incorporate each patient’s information (the researcher used de-identified data). Treatment history was classified according to the definitions provided by the national implementation guideline for MDR-TB control and management and WHO definitions and reporting framework for tuberculosis [3, 4]. New patients are those who have never been treated for TB or have taken anti-TB drugs for less than one month. Relapse patients are those patients who have previously been treated for TB, were declared cured or treatment completed at the end of their most recent course of treatment, and are now diagnosed with a recurrent episode of TB (either a true relapse or a new episode of TB caused by reinfection). Initial treatment failure is a new patient whose smear result was positive at the end of the fifth month or treatment completion. Retreatment failure patients are those who have previously been treated for TB and whose treatment failed at the end of their most recent course of treatment. Smear positive after three months of initial treatment are those new smear positive patients whose smear results are positive at the end of three months of treatment. Return patients are those who are treated for at least one month and interrupt the treatment for more than two months and come back for treatment. Others are those patients who do not fit into any of the above categories.

### Study design and data collection procedure

**Fig 1:**
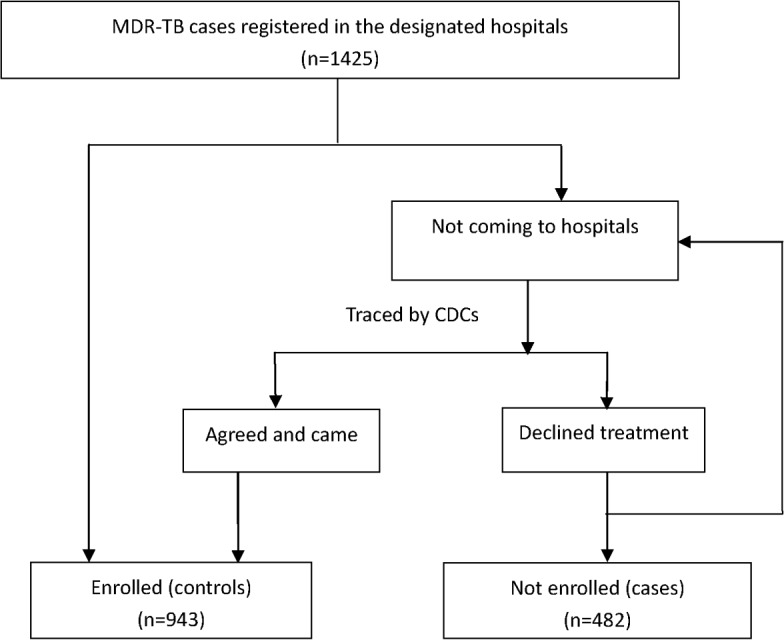
The tracing process for MDR-TB cases.

This study used case-control design. Cases were those patients who were not enrolled to the treatment and controls were those who enrolled to the treatment. The “DST Results Registry form" and “MDR-TB Case registry form” of each patient registered at a designated MDR-TB hospital were reviewed monthly to collect data for diagnosed MDR-TB patients and to identify patients who had not yet enrolled in treatment. TB control workers from each hospital notified the Centers for Disease Control and Prevention (CDC) in the county in which the patients lived. The county CDC then traced these patients with assistance from township doctors and village doctors and completed the "MDR-TB Patient Tracing Registry form". On the registry form, tracing outcomes and the reasons patients had not enrolled in treatment were recorded. These reasons included the following: 1) undergoing economic hardship, 2) out-migrating for work, 3) worrying about work and studies, 4) believing they were cured after receiving drug-sensitive TB treatment, 5) having an unknown address or being unable to be contacted, 6) having died, 7) undergoing non-standard treatment in other hospitals, 8) having other severe diseases or being too weak to undergo MDR-TB treatment, and 9) being migrants from other provinces. Although the patient may have provided many reasons for declining treatment, the most important reason cited for non-enrollment was used to simplify the analysis. The hospital TB control workers also directly called the patients who refused treatment to persuade them to undergo treatment and to verify the tracing information. Hospitals provided monthly information regarding the patients’ enrollment in and initiation of treatment to the county CDC, and the tracing cycle repeated until all patients had been enrolled in treatment (Fig 1). In this study, we analyzed the reason why patients diagnosed with MDR-TB are not enrolled to the treatment hospitals, using the tracing and enrollment information collected up to June 30, 2015.

### Data analysis

All patient forms and clinic records were double entered into a Microsoft Excel 2007 spreadsheet and then imported into SPSS17.0. Odds ratios (ORs) with 95% confidence intervals (CIs) were reported to evaluate the risk factors associated with non-enrollment in MDR-TB treatment in the univariate analyses. All tests were two-tailed, and a P value less than 0.05 was considered statistically significant. Binary logistic regression modeling was used for the multivariate analysis; all variables were initially included in the model, and the forward selection method was used to select variables for inclusion in the final model.

### Ethics statement

Ethical clearance was obtained from the Tuberculosis Research Ethics Review Committee of the Tuberculosis Control Institute of Hunan Province. As this study used secondary data, informed consent was not obtained from each of the study participants. However, the information collected from the secondary data were de-identified (i.e. patients name, phone number, detailed address, and other identifiers of the patients was not included in the data) to maintain confidentiality of the study participants. Pseudonyms were used as a default in all reporting of the research analysis. Measures were taken to protect this confidentiality during the data compilation, storage and analysis. Once the data were collected, it was kept secured.

## Results

### The basic characteristics of MDR-TB patients

In total, 1425 MDR-TB patients were registered in the designated hospitals from 2011 to 2014, of whom 71.9% (1025/1425) were male and 28.1% (400/1425) were female. The patients’ ages ranged from 11 to 85 years old, with a mean age of 44.5±14.8 years. Other data, including residence status, occupation, current living location, and treatment history classification, are shown in Table 1.

**Table 1:** Factors associated with non-enrollment among MDR-TB patients in Hunan province, China.

Characteristics	All cases (N = 1425, 100%) N (col%)	Enrolled (N = 943, 66.2%) N (row%)	Not enrolled (N = 482, 33.8%) N (row%)	COR(95%CI)	AOR(95%CI)
**Sex**					
Male	1025(71.9)	668(65.2)	357(34.8)	Reference	
Female	400(28.1)	275(68.8)	125(31.2)	0.9(0.7–1.1)	
**Age(years)**					
≤20	53(3.7)	37(69.8)	16(30.2)	Reference	Reference
21–40	505(35.4)	362(71.7)	143(28.3)	0.9(0.5–1.7)	0.6(0.3–1.2)
41–60	662(46.5)	452(68.3)	210(31.7)	1.1(0.6–2.0)	0.7(0.3–1.3)
≥61	205(14.4)	92(44.9)	113(55.1)	**2.8(1.5–5.4)**	**2.1(1.0–4.3)**
**Residence status**					
Urban residence	296(20.8)	237(80.1)	59(19.9)	Reference	Reference
Rural residence	1129(79.2)	706(62.5)	423(37.5)	**2.4(1.8–3.3)**	**2.8(1.4–5.4)**
**Occupation**					
City worker	182(12.8)	151(83.0)	31(17.0)	Reference	Reference
Farmer	1059(74.3)	661(62.4)	398(37.6)	**2.9(2.0–4.4)**	1.2(0.6–2.3)
Retired/Student	80(5.6)	58(72.5)	22(27.5)	1.8(1.0–3.5)	0.6(0.3–1.3)
Unemployed/unreported	104(7.3)	73(70.2)	31(29.8)	**2.1(1.2–3.7)**	**2.6(1.4–4.8)**
**Current living location**					
Northern	406(28.5)	269(66.3)	137(33.7)	Reference	
Central	312(21.9)	194(62.2)	118(37.8)	1.2(0.9–1.6)	
Southern	260(18.2)	194(74.6)	66(25.4)	**0.7(0.5–0.9)**	
Southwestern	312(21.9)	212(67.9)	100(32.1)	0.9(0.7–1.3)	
Western	79(5.5)	59(74.7)	20(25.3)	0.7(0.4–1.2)	
Other province	14(1.0)	8(57.1)	6(42.9)	1.5(0.5–4.3)	
Unknown	42(2.9)	7(16.7)	35(83.3)	**9.8(4.3–22.7)**	
**Treatment history classification**					
Retreatment failure	661(46.4)	473(71.6)	188(28.4)	Reference	
New	255(17.9)	144(56.5)	111(43.5)	**1.9(1.4–2.6)**	
Relapse	369(25.9)	232(62.9)	137(37.1)	**1.5(1.1–1.9)**	
Initial treatment failure	103(7.2)	71(68.9)	32(31.1)	1.1(0.7–1.8)	
Smear-positive after 3 months of initial treatment	22(1.5)	15(68.2)	7(31.8)	1.2(0.5–2.9)	
Return or other	15(1.0)	8(53.3)	7(46.7)	2.2(0.8–6.2)	

COR: Crude Odds Ratio; AOR: Adjusted Odds Ratio; CI: Confidence Interval

### The factors associated with non-enrollment among MDR-TB patients

According to “the national implantation guideline for MDR-TB Control and Management” [3], patients diagnosed with MDR-TB should receive standardized in-hospital treatment of two months, to facilitate observation of their condition and the development of an appropriate treatment plan. By the end of 2014, 1425 patients had been diagnosed with MDR-TB, of whom 66.2% (943/1425) and 33.8% (482/1425) had and had not enrolled in standard treatment, respectively. Patients enrolled in treatment were younger than non-enrolled patients (42.8±14.0 years vs 47.9±15.9 years, P<0.05). Based on the results of the univariate analyses, the enrollment rates differed significantly by age group, urban/rural status, occupation, current living location, and treatment history classification. Patients who were 21–40 years old had the highest treatment rate, reaching 71.7%; however, the treatment rate was lowest for patients over the age of 60 years, at 44.9% [OR(95%CI) = 2.8(1.5–5.4)]. Patients from rural areas had a lower enrollment rate than patients from urban areas [OR(95%CI) = 2.4(1.8–3.3)]. Additionally, farmers [OR(95%CI) = 2.9(2.0–4.4)] and unemployed or unreported cases [OR(95%CI) = 2.1(1.2–3.7)] had lower odds of being enrolled in treatment. Patients who had an unknown address or were unable to be contacted had a clearly lower treatment rate [16.7%, OR(95%CI) = 9.8(4.3–22.7)] than patients from the northern part of the province, while southern patients had a higher treatment rate. Regarding treatment history classification, patients registered as “new cases” and “relapse cases” had significantly lower treatment rates than “retreatment failure” cases. No significant gender difference in enrollment was observed. After performing the binary logistic regression analysis, the variables “age≥61 years”, “rural residence”, and “unemployed/unreported” remained in the model, but “farmer”, “southern patients”, “unknown address”, “new case”, and “relapse case” did not (Table 1).

### The reasons for non-enrollment in treatment among MDR-TB patients

According to the tracing results provided byte provincial, county, township and village TB control staff, a total of nine reasons for non-enrollment were reported by the 482 untreated patients. The most frequently reported reason was economic hardship (111 cases), which accounted for 23.0% (111/484) of the reasons reported (Table 2).

**Table 2:** Reasons for non-enrollment in treatment among MDR-TB patients.

Categories	No. of cases	Proportion (%)
Economic hardship	111	23.0
Out-migration for work	87	18.0
Concern about work and studies	66	13.7
Belief of being cured	60	12.4
Death after diagnosis	47	9.8
Non-standard treatment in other hospitals	45	9.3
Unknown address or inability to be contacted	35	7.3
Other severe disease or too weak	25	5.2
Migrants from other provinces	6	1.2
Total	482	100

## Discussion

MDR-TB is characterized by long treatment duration, a high cost of treatment, low cure rates and high mortality rates [5, 6] and is an important infectious source. The level of MDR-TB transmission is directly correlated with the prevalence of primary MDR-TB. Early detection and timely and appropriate treatment are vital to the control of MDR-TB [6]. In this study, 38.2% of diagnosed patients were not enrolled inappropriate treatment; this rate is much higher than the average global rate of 10% but lower than the overall rate of 51% in China [1]. Thus, enrollment in treatment should be prioritized to address the rate of untreated cases as well as the challenges associated with MDR-TB. This study showed that elderly patients (over 60 years old); rural patients; those who were unemployed or had an unreported occupation had lower enrollment rates than their respective counterparts (P<0.05, OR >1).

The low rate of enrollment observed in elderly patients may be related to poor treatment affordability or inability or reluctance to see a doctor; additionally, some elderly patients may lack the confidence to enroll in treatment if they believe that they might not survive for more than a few years regardless of treatment [7]. Rural patients and unemployed patients may be more impoverished and have a higher likelihood of being unable to afford MDR-TB care. As more than 70% of China’s tuberculosis patients are from rural areas, and as rural family incomes were found to be only half of the local average [8], this financial limitation poses a great challenge to treatment affordability.

Economic hardship was the most frequently cited reason for non-enrollment in appropriate treatment. Although the Global Fund Tuberculosis Program and NRCMS may cover almost all direct MDR-TB treatment expenses, patients still have to pay for other related testing fees and may be affected by lost wages, transportation costs and other indirect economic losses. As most MDR-TB patients have been shown to be particularly impoverished, they may choose to not enroll in treatment [9–11], Additional reasons for non-enrollment in treatment are discussed below.

Out-migration for work: Hunan is a province in central China that has substantial population outflow, with many patients being employed in more economically developed areas. These out-migrating patients do not usually return to their hometown to undergo MDR-TB treatment and may not be covered by local health insurance in their workplace. Therefore, it is particularly important to improve the availability of MDR diagnosis and treatment throughout the whole country and provide health security for all migrants [12, 13].

Concerns about work and studies: The full duration of MDR-TB treatment is typically approximately 24 months, and patients need to be hospitalized during the first 1–2 months of treatment [14]. As of the end of 2014, only two hospitals (and predominantly the provincial tuberculosis hospital) had the capacity to properly treat MDR-TB, making treatment costly and inconvenient; therefore, some patients may have terminated treatment because of worries related to the effects of the treatment’s long duration and of hospitalization on their work and studies [15].

Belief that they are cured: Since rapid molecular testing methods were not widely used in this setting during the study period, most cases of MDR-TB were detected using routine phenotypic drug susceptibility testing, including the collection of sputum samples for culture (solid or liquid), identification of MTB  and performance of drug susceptibility testing; this testing process is lengthy, often requiring 2–3 months [16]. After the final results of these tests became available, the patients had often left the hospital where they had been originally diagnosed [17, 18]. Additionally, some patients may have previously completed the 6–8 months of antituberculosis treatment may not have had obvious symptoms, causing them to believe that they had been cured and to decline further MDR-TB treatment [19, 20].

Death after diagnosis: The results of this study clearly illustrate the high mortality rate associated with MDR-TB. Higher mortality rates have previously been reported in patients in China who have not initiated treatment compared to those who have enrolled in MDR-TB treatment [21, 22], suggesting that early detection and early treatment are extremely important in reducing the mortality rates associated with this disease.

Non-standard treatment in other hospitals: MDR-TB treatment is a systematic process that requires skilled clinical experts, advanced diagnosis equipment, a sufficient drug supply and strict adherence to guidelines; accordingly, only two hospitals were designated to provide MDR-TB treatment during the study period. Some patients may have had low levels of awareness of the importance of standardized treatment and thus undergone improper treatment in other hospitals; consequently, these patients may have been more difficult to cure, facilitating the spread of the disease [23].

Unknown address or inability to be contacted:TB patients may be reluctant to provide detailed addresses during treatment, and in this study, some patients provided fake telephone numbers, fearing potential disclosure of their personal health information; this fear may have resulted in them being lost to follow-up. This finding suggests that medical personnel should be more patient when asking for personal information from patients and maintain the confidentiality of collected data to mitigate these patients’ concerns [24].

### Limitations

This study mainly focused on the process of tracing patients from MDR-TB detection to enrollment in treatment, and the detection rates and treatment success rates were not assessed. Thus, these data may not reflect the overall effects of MDR-TB control in Hunan Province. Additionally, the reasons for which patients had not enrolled in MDR-TB treatment were often very complex and might be due to a combination of factors. However, to simplify this study’s analyses, only the most important reason cited was recorded for each patient. An information bias could also have existed due to investigator misunderstanding. However, due to this study’s large sample size and long-term follow-up, the reasons for non-enrollment in treatment in these patients should be largely reflected in the findings.

## Conclusion

Elderly patients, rural patients, unemployed or occupation unreported patients were more likely to decline treatment than their respective counterparts. The leading cause of non-enrollment in MDR-TB treatment was economic hardship; however, out-migrating for work and worrying about work and studies were also cited as important barriers to enrollment. Therefore, comprehensive strategies targeting priority populations, especially those enhancing treatment affordability and availability, need to be implemented to improve MDR-TB control.

## Supporting Information

S1 Table Factors and reasons for enrollment among MDR-TB cases. (PDF)
