# Supplementary material for: Reasons for Non-Enrollment in Treatment among Multi-Drug Resistant Tuberculosis Patients in Hunan Province, China
Source: PLoS One. 2017 Jan 23;12(1):e0170718. doi: 10.1371/journal.pone.0170718 (PMC5257000; doi:10.1371/journal.pone.0170718)
Supplement: S1 Table — (PDF) [file pone.0170718.s001.pdf]

Patient number	Sex	Age	Resid	Location	Occupation	Treatment history classification	Enrollment	Reasons for non-enrollment
1	Male	51	Urban	Northern	City worker	Relapse	Enrolled	
2	Femal	43	Rural	Northern	Farmer	Retreatment failure	Enrolled	
3	Femal	50	Urban	Northern	Unemployed/unreported	Retreatment failure	Enrolled	
4	Male	31	Rural	Unknown	Farmer	New	Not enrolled	Unknown address
5	Male	64	Rural	Northern	Farmer	Retreatment failure	Not enrolled	Death
6	Male	36	Rural	Other province	Farmer	Retreatment failure	Not enrolled	Migrants from other provinces
7	Male	52	Urban	Central	City worker	Relapse	Enrolled	
8	Femal	28	Rural	Northern	Farmer	Retreatment failure	Not enrolled	Non-standard treatment
9	Male	46	Rural	Northern	Farmer	Relapse	Enrolled	
10	Male	50	Rural	Northern	Farmer	Retreatment failure	Enrolled	
11	Male	47	Rural	Central	Farmer	New	Enrolled	
12	Male	50	Rural	Northern	Farmer	New	Not enrolled	Out-migration
13	Male	42	Rural	Central	Farmer	Relapse	Enrolled	
14	Male	60	Rural	Central	Farmer	Smear+ after 3 ms	Enrolled	
15	Male	60	Rural	Southern	Farmer	New	Enrolled	
16	Male	42	Rural	Southern	Farmer	Retreatment failure	Not enrolled	Out-migration
17	Male	54	Rural	Southern	Farmer	Relapse	Not enrolled	Economic hardship
18	Male	37	Rural	Southern	Farmer	Retreatment failure	Enrolled	
19	Male	30	Rural	Western	Farmer	Retreatment failure	Enrolled	
20	Male	26	Rural	Southern	Farmer	Retreatment failure	Enrolled	
21	Male	55	Rural	Southern	Farmer	Retreatment failure	Not enrolled	Death
22	Male	34	Rural	Other province	Farmer	Initial treatment failur	Not enrolled	Migrants from other provinces
23	Male	64	Rural	Western	Farmer	New	Enrolled	
24	Femal	23	Rural	Southwestern	Farmer	Retreatment failure	Enrolled	
25	Femal	74	Rural	Southwestern	Farmer	Retreatment failure	Enrolled	
26	Male	44	Rural	Northern	Farmer	New	Enrolled	
27	Male	66	Rural	Northern	Farmer	Retreatment failure	Not enrolled	Death
28	Male	49	Urban	Central	City worker	New	Enrolled	
29	Male	25	Rural	Northern	Farmer	New	Enrolled	
30	Femal	19	Rural	Southwestern	Farmer	New	Not enrolled	studies
31	Male	62	Rural	Central	Farmer	Relapse	Not enrolled	Economic hardship
32	Male	50	Rural	Central	Farmer	New	Enrolled	
33	Male	59	Rural	Central	Farmer	Relapse	Not enrolled	Economic hardship
34	Male	51	Rural	Northern	Farmer	Return or other	Not enrolled	Economic hardship
35	Femal	38	Urban	Central	City worker	Retreatment failure	Enrolled	
36	Male	35	Urban	Western	City worker	Retreatment failure	Enrolled	
37	Male	65	Rural	Northern	Farmer	Relapse	Not enrolled	Economic hardship
38	Male	57	Rural	Southern	Farmer	Retreatment failure	Not enrolled	Economic hardship
39	Male	41	Rural	Southwestern	Farmer	Retreatment failure	Enrolled	
40	Femal	39	Urban	Unknown	City worker	Retreatment failure	Not enrolled	Unknown address
41	Femal	47	Rural	Southern	Farmer	Relapse	Enrolled	
42	Male	60	Rural	Unknown	City worker	New	Not enrolled	Unknown address
43	Femal	32	Rural	Western	Farmer	Retreatment failure	Enrolled	
44	Femal	61	Rural	Central	Farmer	Relapse	Not enrolled	Economic hardship
45	Male	48	Rural	Northern	Farmer	Return or other	Not enrolled	Out-migration
46	Male	39	Rural	Northern	Farmer	Relapse	Enrolled	
47	Male	26	Rural	Southern	Farmer	Relapse	Enrolled	
48	Femal	28	Urban	Unknown	City worker	Relapse	Not enrolled	Unknown address
49	Male	38	Rural	Southwestern	City worker	Retreatment failure	Not enrolled	Out-migration
50	Male	42	Urban	Unknown	City worker	Retreatment failure	Not enrolled	Unknown address
51	Femal	48	Rural	Southwestern	Farmer	Relapse	Enrolled	
52	Male	38	Rural	Central	Farmer	Relapse	Enrolled	
53	Femal	25	Rural	Southern	Farmer	Initial treatment failur	Enrolled	
54	Male	34	Rural	Central	Farmer	Retreatment failure	Enrolled	
55	Femal	31	Urban	Central	City worker	Relapse	Not enrolled	studies
56	Male	49	Rural	Northern	Farmer	Relapse	Not enrolled	Economic hardship
57	Male	41	Rural	Southern	Farmer	Retreatment failure	Enrolled	
58	Male	44	Rural	Central	Farmer	New	Enrolled	

59	Male	54	Rural	Central	Farmer	Relapse	Enrolled	
60	Male	42	Rural	Central	Farmer	Retreatment failure	Enrolled	
61	Femal	33	Rural	Southern	Farmer	Retreatment failure	Enrolled	
62	Femal	58	Rural	Central	Farmer	New	Enrolled	
63	Male	48	Rural	Southwestern	Farmer	Retreatment failure	Enrolled	
64	Male	59	Rural	Unknown	City worker	Initial treatment failur	Not enrolled	Unknown address
65	Male	34	Rural	Central	Farmer	Initial treatment failur	Enrolled	
66	Femal	29	Rural	Central	City worker	Smear+ after 3 ms	Not enrolled	Non-standard treatment
67	Femal	45	Rural	Northern	Farmer	Relapse	Not enrolled	Out-migration
68	Femal	28	Urban	Southwestern	Unemployed/unreported	Relapse	Not enrolled	Death
69	Femal	78	Urban	Central	City worker	Retreatment failure	Not enrolled	Other severe disease
70	Femal	49	Rural	Southern	City worker	Retreatment failure	Not enrolled	studies
71	Femal	34	Urban	Central	City worker	Retreatment failure	Enrolled	
72	Femal	53	Rural	Northern	Farmer	Retreatment failure	Enrolled	
73	Male	54	Rural	Unknown	Farmer	Retreatment failure	Enrolled	
74	Male	60	Rural	Northern	Farmer	New	Enrolled	
75	Male	31	Rural	Northern	Farmer	New	Enrolled	
76	Male	58	Rural	Southern	Farmer	Relapse	Enrolled	
77	Male	61	Urban	Southern	Retired/Student	Relapse	Enrolled	
78	Male	61	Rural	Central	Farmer	Smear+ after 3 ms	Enrolled	
79	Male	36	Rural	Southern	Farmer	Retreatment failure	Enrolled	
80	Male	36	Rural	Southwestern	Farmer	Retreatment failure	Not enrolled	Concern about work and studies
81	Male	25	Rural	Central	Farmer	Relapse	Enrolled	
82	Male	52	Rural	Southern	Farmer	Retreatment failure	Enrolled	
83	Male	20	Urban	Central	City worker	Retreatment failure	Not enrolled	Concern about work and studies
84	Male	29	Rural	Central	Farmer	Retreatment failure	Not enrolled	Non-standard treatment
85	Femal	62	Rural	Southwestern	City worker	Retreatment failure	Not enrolled	studies
86	Male	32	Rural	Central	Farmer	Retreatment failure	Enrolled	
87	Male	24	Rural	Northern	Farmer	Retreatment failure	Enrolled	
88	Male	47	Rural	Southwestern	Farmer	Retreatment failure	Enrolled	
89	Male	45	Urban	Western	Unemployed/unreported	Initial treatment failur	Enrolled	
90	Male	58	Rural	Southwestern	Farmer	Retreatment failure	Not enrolled	Death
91	Male	75	Rural	Northern	Farmer	Relapse	Not enrolled	Economic hardship
92	Male	57	Urban	Northern	Unemployed/unreported	Return or other	Not enrolled	studies
93	Male	57	Rural	Northern	Farmer	Retreatment failure	Enrolled	
94	Male	53	Rural	Northern	Farmer	Relapse	Enrolled	
95	Male	19	Urban	Central	Unemployed/unreported	New	Not enrolled	studies
96	Male	46	Rural	Southern	Farmer	Retreatment failure	Enrolled	
97	Male	43	Rural	Northern	Farmer	Relapse	Enrolled	
98	Femal	23	Rural	Other province	Farmer	Retreatment failure	Enrolled	
99	Male	63	Rural	Northern	Retired/Student	Relapse	Enrolled	
100	Femal	27	Rural	Northern	Farmer	Retreatment failure	Enrolled	
101	Male	75	Urban	Central	City worker	Retreatment failure	Not enrolled	Other severe disease
102	Male	77	Urban	Central	Retired/Student	Relapse	Enrolled	
103	Male	74	Rural	Northern	Farmer	Retreatment failure	Not enrolled	Other severe disease
104	Male	51	Rural	Central	Farmer	Relapse	Enrolled	
105	Male	30	Urban	Central	City worker	Relapse	Not enrolled	studies
106	Male	30	Rural	Southwestern	Farmer	Initial treatment failur	Enrolled	
107	Male	29	Rural	Northern	Farmer	New	Not enrolled	Economic hardship
108	Femal	19	Urban	Southwestern	Retired/Student	Relapse	Enrolled	
109	Femal	61	Urban	Southwestern	Retired/Student	Relapse	Enrolled	
110	Male	55	Urban	Unknown	Unemployed/unreported	New	Not enrolled	Unknown address
111	Male	37	Rural	Northern	Farmer	Relapse	Enrolled	
112	Femal	51	Rural	Northern	Farmer	Relapse	Enrolled	
113	Femal	49	Rural	Southern	Farmer	Initial treatment failur	Enrolled	
114	Male	55	Rural	Northern	City worker	Retreatment failure	Not enrolled	Belief of being cured
115	Male	64	Rural	Central	Farmer	Relapse	Enrolled	
116	Male	44	Urban	Northern	City worker	Retreatment failure	Enrolled	
117	Femal	40	Rural	Southwestern	Farmer	Initial treatment failur	Enrolled	
118	Male	31	Rural	Southern	City worker	Relapse	Not enrolled	studies
119	Male	62	Rural	Northern	City worker	Initial treatment failur	Not enrolled	studies

120	Male	77	Rural	Southwestern	Farmer	Retreatment failure	Enrolled	
121	Male	38	Rural	Southwestern	Farmer	Retreatment failure	Enrolled	
122	Femal	48	Rural	Central	Farmer	Retreatment failure	Enrolled	
123	Femal	23	Rural	Southern	Farmer	Relapse	Enrolled	
124	Male	66	Rural	Southern	Retired/Student	Retreatment failure	Enrolled	
125	Male	38	Rural	Southwestern	Farmer	Relapse	Enrolled	
126	Male	63	Rural	Northern	Farmer	Initial treatment failur	Enrolled	
127	Femal	48	Rural	Northern	Farmer	Retreatment failure	Enrolled	
128	Femal	29	Rural	Western	Farmer	Retreatment failure	Enrolled	
129	Male	19	Rural	Northern	City worker	Relapse	Not enrolled	studies
130	Male	22	Rural	Central	Farmer	New	Enrolled	
131	Male	24	Rural	Northern	City worker	Retreatment failure	Not enrolled	studies
132	Male	26	Urban	Northern	City worker	Smear+ after 3 ms	Enrolled	
133	Femal	39	Rural	Western	City worker	Retreatment failure	Not enrolled	Belief of being cured
134	Femal	45	Rural	Western	Farmer	Retreatment failure	Enrolled	
135	Male	40	Rural	Southern	Farmer	Initial treatment failur	Enrolled	
136	Male	11	Urban	Southern	Retired/Student	Retreatment failure	Not enrolled	Non-standard treatment
137	Male	53	Rural	Northern	City worker	Retreatment failure	Not enrolled	studies
138	Male	60	Rural	Central	Farmer	Relapse	Not enrolled	Economic hardship
139	Femal	50	Rural	Southern	Farmer	Smear+ after 3 ms	Enrolled	
140	Male	38	Rural	Western	Farmer	Retreatment failure	Enrolled	
141	Femal	19	Urban	Central	Retired/Student	Initial treatment failur	Enrolled	
142	Male	39	Rural	Southern	Farmer	New	Enrolled	
143	Male	28	Rural	Southern	Farmer	New	Enrolled	
144	Male	43	Rural	Central	Farmer	Relapse	Not enrolled	Concern about work and studies
145	Femal	24	Rural	Central	Farmer	New	Enrolled	
146	Male	39	Rural	Southwestern	Farmer	Retreatment failure	Not enrolled	Non-standard treatment
147	Male	58	Rural	Northern	City worker	Retreatment failure	Not enrolled	studies
148	Male	45	Rural	Central	Farmer	New	Not enrolled	Economic hardship
149	Male	21	Rural	Northern	Farmer	Retreatment failure	Enrolled	
150	Male	38	Rural	Northern	Farmer	Retreatment failure	Enrolled	
151	Femal	36	Urban	Southwestern	City worker	Retreatment failure	Not enrolled	studies
152	Male	31	Rural	Western	Farmer	Relapse	Enrolled	
153	Male	21	Rural	Unknown	Farmer	Retreatment failure	Enrolled	
154	Male	21	Rural	Northern	Farmer	Retreatment failure	Enrolled	
155	Femal	53	Rural	Northern	Farmer	Retreatment failure	Enrolled	
156	Male	60	Rural	Southern	Farmer	New	Enrolled	
157	Male	47	Rural	Central	Farmer	Retreatment failure	Enrolled	
158	Male	42	Rural	Northern	City worker	Relapse	Not enrolled	studies
159	Femal	54	Rural	Northern	Farmer	Retreatment failure	Enrolled	
160	Male	37	Rural	Northern	Farmer	Retreatment failure	Enrolled	
161	Femal	30	Rural	Southwestern	Farmer	Retreatment failure	Enrolled	
162	Femal	49	Rural	Central	Farmer	Initial treatment failur	Enrolled	
163	Male	43	Rural	Central	Farmer	New	Not enrolled	Economic hardship
164	Femal	51	Rural	Southern	Farmer	Relapse	Not enrolled	Death
165	Male	42	Urban	Southern	City worker	Return or other	Enrolled	
166	Male	71	Rural	Southwestern	Retired/Student	Relapse	Enrolled	
167	Male	43	Rural	Northern	Farmer	Retreatment failure	Enrolled	
168	Femal	20	Rural	Central	Farmer	New	Enrolled	
169	Femal	41	Rural	Northern	Farmer	Retreatment failure	Enrolled	
170	Male	50	Rural	Northern	Farmer	Relapse	Enrolled	
171	Male	71	Rural	Southwestern	Farmer	Relapse	Enrolled	
172	Male	67	Urban	Southwestern	City worker	Retreatment failure	Not enrolled	Concern about work and studies
173	Male	47	Rural	Southwestern	Farmer	Relapse	Enrolled	
174	Femal	36	Rural	Northern	Farmer	Retreatment failure	Not enrolled	Economic hardship
175	Femal	47	Rural	Western	Farmer	Retreatment failure	Enrolled	
176	Male	23	Rural	Northern	Farmer	Initial treatment failur	Enrolled	
177	Femal	19	Rural	Northern	Farmer	Relapse	Not enrolled	Economic hardship
178	Male	68	Rural	Northern	Farmer	Relapse	Not enrolled	Economic hardship
179	Male	67	Rural	Northern	Farmer	Retreatment failure	Not enrolled	Economic hardship
180	Male	61	Rural	Southern	Farmer	Retreatment failure	Not enrolled	Out-migration

181	Male	57	Urban	Northern	City worker	Retreatment failure	Enrolled	
182	Male	57	Rural	Central	Farmer	New	Not enrolled	Economic hardship
183	Femal	53	Urban	Unknown	Unemployed/unreported	Retreatment failure	Not enrolled	Unknown address
184	Femal	49	Rural	Northern	Farmer	Retreatment failure	Enrolled	
185	Femal	36	Rural	Northern	Farmer	Retreatment failure	Enrolled	
186	Male	63	Rural	Central	Farmer	Retreatment failure	Not enrolled	studies
187	Male	49	Rural	Northern	Farmer	Retreatment failure	Not enrolled	Out-migration
188	Male	56	Urban	Unknown	City worker	Retreatment failure	Not enrolled	Unknown address
189	Male	17	Rural	Northern	Retired/Student	Initial treatment failur	Enrolled	
190	Femal	26	Rural	Northern	Farmer	Retreatment failure	Enrolled	
191	Male	19	Urban	Southwestern	Retired/Student	Retreatment failure	Enrolled	
192	Male	63	Rural	Northern	Retired/Student	Relapse	Enrolled	
193	Male	63	Rural	Northern	Farmer	New	Enrolled	
194	Femal	17	Rural	Central	Retired/Student	Retreatment failure	Enrolled	
195	Male	32	Urban	Unknown	City worker	Retreatment failure	Not enrolled	Unknown address
196	Male	20	Rural	Southern	Farmer	New	Enrolled	
197	Male	38	Rural	Southern	Farmer	Retreatment failure	Enrolled	
198	Male	48	Rural	Western	Farmer	Relapse	Enrolled	
199	Male	55	Rural	Southwestern	Farmer	Retreatment failure	Enrolled	
200	Femal	59	Rural	Southern	Farmer	New	Enrolled	
201	Male	22	Rural	Northern	Farmer	New	Not enrolled	Belief of being cured
202	Male	18	Rural	Northern	Farmer	Initial treatment failur	Not enrolled	studies
203	Femal	51	Rural	Northern	Farmer	Retreatment failure	Not enrolled	Belief of being cured
204	Male	41	Rural	Northern	Farmer	Retreatment failure	Enrolled	
205	Male	38	Rural	Northern	Farmer	Retreatment failure	Enrolled	
206	Male	56	Rural	Southern	Farmer	Initial treatment failur	Enrolled	
207	Male	61	Rural	Southwestern	Farmer	Relapse	Enrolled	
208	Male	56	Rural	Southwestern	Farmer	Relapse	Enrolled	
209	Male	54	Rural	Central	Farmer	Retreatment failure	Enrolled	
210	Male	49	Urban	Central	Unemployed/unreported	Relapse	Enrolled	
211	Male	76	Rural	Southwestern	Farmer	New	Not enrolled	Other severe disease
212	Femal	53	Rural	Northern	Farmer	Relapse	Enrolled	
213	Male	25	Rural	Southwestern	Farmer	Retreatment failure	Not enrolled	Economic hardship
214	Femal	42	Urban	Northern	City worker	Retreatment failure	Enrolled	
215	Femal	60	Rural	Southern	Farmer	Relapse	Enrolled	
216	Male	75	Rural	Central	Farmer	Retreatment failure	Not enrolled	Economic hardship
217	Femal	31	Rural	Southwestern	Farmer	Retreatment failure	Enrolled	
218	Male	31	Urban	Southern	City worker	Initial treatment failur	Enrolled	
219	Femal	28	Urban	Unknown	Unemployed/unreported	Retreatment failure	Not enrolled	Unknown address
220	Femal	60	Rural	Northern	Farmer	Retreatment failure	Not enrolled	Economic hardship
221	Femal	60	Rural	Central	Farmer	Relapse	Enrolled	
222	Male	48	Rural	Central	Farmer	Relapse	Enrolled	
223	Male	59	Rural	Central	Farmer	Relapse	Enrolled	
224	Male	40	Rural	Southern	Farmer	Retreatment failure	Enrolled	
225	Femal	44	Rural	Unknown	Farmer	Relapse	Enrolled	
226	Male	50	Urban	Southern	City worker	Retreatment failure	Enrolled	
227	Femal	69	Rural	Southwestern	Farmer	Relapse	Enrolled	
228	Femal	51	Rural	Central	Farmer	Relapse	Enrolled	
229	Male	60	Rural	Southern	Farmer	Retreatment failure	Not enrolled	Economic hardship
230	Male	58	Rural	Southwestern	Farmer	Relapse	Enrolled	
231	Male	31	Rural	Southern	Farmer	Retreatment failure	Enrolled	
232	Femal	78	Rural	Unknown	Farmer	Retreatment failure	Not enrolled	Unknown address
233	Male	31	Rural	Central	Farmer	Relapse	Not enrolled	Economic hardship
234	Male	30	Rural	Northern	Farmer	Retreatment failure	Enrolled	
235	Femal	29	Rural	Northern	Farmer	Relapse	Not enrolled	Economic hardship
236	Male	42	Rural	Southern	Farmer	Retreatment failure	Not enrolled	Out-migration
237	Male	52	Rural	Southern	Farmer	Relapse	Not enrolled	Economic hardship
238	Male	72	Rural	Central	Farmer	Initial treatment failur	Enrolled	
239	Male	39	Rural	Northern	Farmer	Retreatment failure	Not enrolled	Belief of being cured
240	Male	62	Urban	Central	Retired/Student	Retreatment failure	Enrolled	
241	Femal	55	Rural	Northern	Farmer	Relapse	Enrolled	

242	Male	23	Rural	Central	Farmer	Retreatment failure	Not enrolled	Economic hardship
243	Femal	53	Rural	Northern	Farmer	Initial treatment failur	Enrolled	
244	Male	56	Rural	Southwestern	Farmer	Retreatment failure	Not enrolled	Economic hardship
245	Male	44	Rural	Southwestern	Farmer	Retreatment failure	Enrolled	
246	Male	52	Rural	Southern	Farmer	Smear+ after 3 ms	Enrolled	
247	Male	37	Urban	Northern	City worker	Relapse	Enrolled	
248	Male	60	Urban	Southwestern	Unemployed/unreported	Retreatment failure	Enrolled	
249	Male	25	Urban	Southern	City worker	Relapse	Enrolled	
250	Male	57	Urban	Northern	City worker	Retreatment failure	Enrolled	
251	Male	78	Rural	Central	Farmer	Initial treatment failur	Enrolled	
252	Male	18	Rural	Southwestern	Retired/Student	Retreatment failure	Enrolled	
253	Male	51	Rural	Northern	Farmer	New	Enrolled	
254	Femal	44	Rural	Northern	Farmer	Relapse	Enrolled	
255	Femal	33	Rural	Central	Farmer	Retreatment failure	Enrolled	
256	Male	61	Rural	Southwestern	Farmer	New	Enrolled	
257	Male	62	Rural	Southern	Farmer	Retreatment failure	Enrolled	
258	Femal	40	Rural	Southern	Farmer	Relapse	Enrolled	
259	Male	42	Rural	Central	Farmer	Retreatment failure	Enrolled	
260	Male	41	Rural	Central	Farmer	Retreatment failure	Not enrolled	Death
261	Male	54	Rural	Northern	Farmer	Relapse	Enrolled	
262	Male	28	Urban	Northern	City worker	New	Enrolled	
263	Femal	53	Rural	Western	Farmer	Retreatment failure	Not enrolled	Non-standard treatment
264	Male	30	Rural	Southwestern	Farmer	Relapse	Enrolled	
265	Male	45	Rural	Southwestern	Farmer	Retreatment failure	Not enrolled	Out-migration
266	Male	44	Rural	Northern	Farmer	Retreatment failure	Enrolled	
267	Femal	43	Rural	Southwestern	Farmer	Retreatment failure	Not enrolled	studies
268	Male	32	Rural	Western	Farmer	Retreatment failure	Enrolled	
269	Male	57	Rural	Northern	Farmer	Relapse	Enrolled	
270	Male	54	Rural	Southern	Farmer	Relapse	Enrolled	
271	Male	41	Rural	Central	Farmer	Retreatment failure	Enrolled	
272	Male	44	Urban	Southern	City worker	Retreatment failure	Enrolled	
273	Male	62	Rural	Southern	Farmer	Relapse	Not enrolled	Belief of being cured
274	Male	37	Rural	Southern	Farmer	New	Not enrolled	Out-migration
275	Male	37	Rural	Northern	Farmer	Retreatment failure	Enrolled	
276	Femal	43	Rural	Northern	Farmer	Retreatment failure	Enrolled	
277	Femal	32	Rural	Southern	Farmer	Retreatment failure	Enrolled	
278	Male	38	Rural	Southern	Farmer	Retreatment failure	Enrolled	
279	Male	23	Urban	Southwestern	Unemployed/unreported	Retreatment failure	Enrolled	
280	Femal	21	Urban	Northern	Unemployed/unreported	Retreatment failure	Enrolled	
281	Male	41	Rural	Western	Farmer	Retreatment failure	Enrolled	
282	Male	70	Rural	Northern	Farmer	Relapse	Not enrolled	Economic hardship
283	Male	47	Rural	Southern	Farmer	Relapse	Enrolled	
284	Femal	63	Rural	Central	Farmer	Retreatment failure	Enrolled	
285	Femal	19	Urban	Southwestern	Retired/Student	Retreatment failure	Not enrolled	Non-standard treatment
286	Male	71	Rural	Northern	Farmer	New	Enrolled	
287	Male	40	Rural	Southern	Farmer	Retreatment failure	Enrolled	
288	Male	49	Urban	Southern	Unemployed/unreported	Retreatment failure	Enrolled	
289	Femal	44	Urban	Central	Unemployed/unreported	Retreatment failure	Enrolled	
290	Male	43	Rural	Western	Farmer	Relapse	Enrolled	
291	Male	78	Rural	Northern	Farmer	New	Enrolled	
292	Femal	43	Urban	Southwestern	Unemployed/unreported	Retreatment failure	Enrolled	
293	Male	27	Rural	Southwestern	Farmer	Retreatment failure	Not enrolled	Economic hardship
294	Male	46	Rural	Southwestern	Farmer	Relapse	Not enrolled	Concern about work and studies
295	Male	59	Rural	Central	Farmer	Retreatment failure	Not enrolled	Non-standard treatment
296	Male	22	Rural	Southwestern	Farmer	New	Not enrolled	Economic hardship
297	Femal	42	Rural	Southern	Farmer	Relapse	Enrolled	
298	Male	47	Rural	Northern	Farmer	Retreatment failure	Not enrolled	Belief of being cured
299	Male	44	Urban	Southern	Unemployed/unreported	Retreatment failure	Enrolled	
300	Male	61	Rural	Central	Farmer	Retreatment failure	Enrolled	
301	Male	49	Rural	Southern	Farmer	Retreatment failure	Enrolled	
302	Femal	41	Rural	Southern	Farmer	Retreatment failure	Not enrolled	Concern about work and studies

303	Male	64	Rural	Central	Farmer	Relapse	Enrolled	
304	Femal	51	Rural	Southern	Farmer	Retreatment failure	Not enrolled	Economic hardship
305	Femal	27	Rural	Central	Farmer	Retreatment failure	Enrolled	
306	Male	48	Rural	Northern	Farmer	New	Enrolled	
307	Male	49	Rural	Southwestern	Farmer	Retreatment failure	Not enrolled	Economic hardship
308	Femal	32	Rural	Southern	Farmer	Relapse	Enrolled	
309	Femal	58	Rural	Southern	Farmer	New	Enrolled	
310	Male	62	Rural	Central	Farmer	Smear+ after 3 ms	Enrolled	
311	Male	53	Rural	Western	Farmer	Retreatment failure	Enrolled	
312	Femal	27	Rural	Central	Farmer	Initial treatment failur	Enrolled	
313	Femal	23	Urban	Southern	City worker	Retreatment failure	Enrolled	
314	Male	32	Urban	Southern	City worker	Retreatment failure	Enrolled	
315	Femal	32	Rural	Southwestern	Farmer	Relapse	Enrolled	
316	Male	80	Rural	Northern	Farmer	Relapse	Not enrolled	Economic hardship
317	Femal	49	Urban	Southwestern	Unemployed/unreported	Retreatment failure	Not enrolled	Out-migration
318	Male	43	Urban	Southwestern	Unemployed/unreported	Retreatment failure	Enrolled	
319	Male	67	Urban	Southwestern	Retired/Student	Relapse	Enrolled	
320	Male	66	Rural	Central	Farmer	Retreatment failure	Not enrolled	Economic hardship
321	Male	54	Rural	Northern	Farmer	Retreatment failure	Not enrolled	studies
322	Male	74	Rural	Central	Farmer	Relapse	Not enrolled	Other severe disease
323	Male	50	Rural	Northern	Farmer	Retreatment failure	Enrolled	
324	Femal	48	Rural	Northern	Farmer	Return or other	Enrolled	
325	Femal	49	Rural	Northern	Farmer	Initial treatment failur	Enrolled	
326	Femal	77	Rural	Northern	Farmer	Retreatment failure	Not enrolled	Economic hardship
327	Male	80	Rural	Northern	Farmer	Retreatment failure	Not enrolled	Other severe disease
328	Male	22	Rural	Unknown	Farmer	Retreatment failure	Enrolled	
329	Femal	18	Rural	Central	Retired/Student	Retreatment failure	Not enrolled	Concern about work and studies
330	Male	40	Rural	Southwestern	Farmer	Retreatment failure	Enrolled	
331	Femal	27	Rural	Northern	Farmer	Retreatment failure	Enrolled	
332	Male	55	Rural	Northern	Farmer	Relapse	Enrolled	
333	Male	50	Urban	Northern	City worker	Relapse	Enrolled	
334	Male	66	Rural	Central	Farmer	Return or other	Not enrolled	Economic hardship
335	Male	50	Rural	Northern	Farmer	Retreatment failure	Not enrolled	Non-standard treatment
336	Male	51	Rural	Southern	Farmer	Retreatment failure	Enrolled	
337	Male	22	Rural	Central	Farmer	Relapse	Enrolled	
338	Male	51	Urban	Central	Unemployed/unreported	Relapse	Enrolled	
339	Femal	43	Rural	Northern	Farmer	Retreatment failure	Enrolled	
340	Femal	60	Rural	Central	Farmer	Relapse	Enrolled	
341	Male	60	Rural	Northern	Unemployed/unreported	Retreatment failure	Enrolled	
342	Male	38	Rural	Central	Farmer	Relapse	Enrolled	
343	Male	70	Rural	Southern	Farmer	New	Not enrolled	Economic hardship
344	Male	55	Rural	Southern	Farmer	Relapse	Enrolled	
345	Male	26	Rural	Central	Farmer	Retreatment failure	Not enrolled	Out-migration
346	Femal	28	Rural	Northern	Farmer	New	Not enrolled	Out-migration
347	Male	37	Rural	Southern	Unemployed/unreported	Retreatment failure	Enrolled	
348	Male	48	Rural	Southern	Farmer	Retreatment failure	Enrolled	
349	Male	58	Rural	Southern	Farmer	New	Enrolled	
350	Male	50	Rural	Southern	Farmer	Retreatment failure	Not enrolled	Non-standard treatment
351	Femal	36	Rural	Southwestern	Farmer	Retreatment failure	Not enrolled	Out-migration
352	Femal	45	Rural	Southern	Farmer	Relapse	Enrolled	
353	Male	67	Rural	Southern	Farmer	Retreatment failure	Not enrolled	Other severe disease
354	Male	48	Rural	Northern	Farmer	Initial treatment failur	Not enrolled	Out-migration
355	Male	44	Rural	Northern	Farmer	Retreatment failure	Enrolled	
356	Male	61	Rural	Central	Farmer	Relapse	Not enrolled	Out-migration
357	Male	43	Rural	Southern	Farmer	Retreatment failure	Enrolled	
358	Male	41	Rural	Southern	Farmer	Retreatment failure	Enrolled	
359	Femal	54	Urban	Northern	Unemployed/unreported	New	Not enrolled	studies
360	Male	33	Rural	Southern	Farmer	Retreatment failure	Not enrolled	Non-standard treatment
361	Male	43	Rural	Southwestern	Farmer	Retreatment failure	Enrolled	
362	Femal	22	Urban	Southern	City worker	New	Enrolled	
363	Male	38	Rural	Northern	Farmer	Retreatment failure	Enrolled	

364	Male	54	Rural	Northern	Farmer	New	Enrolled	
365	Femal	29	Urban	Southwestern	City worker	Relapse	Enrolled	
366	Femal	31	Rural	Northern	Farmer	Retreatment failure	Enrolled	
367	Male	46	Rural	Central	Farmer	Retreatment failure	Enrolled	
368	Male	31	Rural	Southwestern	Farmer	New	Enrolled	
369	Femal	58	Rural	Central	Farmer	New	Not enrolled	Out-migration
370	Male	62	Rural	Northern	Farmer	New	Not enrolled	Economic hardship
371	Male	54	Urban	Southwestern	City worker	Relapse	Enrolled	
372	Male	48	Rural	Southern	Farmer	Relapse	Enrolled	
373	Male	15	Rural	Southern	Retired/Student	Retreatment failure	Enrolled	
374	Male	63	Rural	Southern	Farmer	Retreatment failure	Enrolled	
375	Male	25	Urban	Northern	City worker	Relapse	Enrolled	
376	Male	32	Rural	Southern	Farmer	Retreatment failure	Enrolled	
377	Male	55	Rural	Northern	Farmer	Smear+ after 3 ms	Enrolled	
378	Male	59	Urban	Northern	City worker	Relapse	Enrolled	
379	Male	21	Urban	Central	Unemployed/unreported	Relapse	Enrolled	
380	Male	20	Rural	Central	Retired/Student	Retreatment failure	Not enrolled	Concern about work and studies
381	Male	50	Rural	Northern	Farmer	Retreatment failure	Enrolled	
382	Male	25	Rural	Northern	Farmer	Relapse	Enrolled	
383	Male	60	Rural	Central	Farmer	Retreatment failure	Not enrolled	Out-migration
384	Femal	51	Rural	Northern	Farmer	Retreatment failure	Enrolled	
385	Femal	32	Rural	Southern	Farmer	Initial treatment failur	Not enrolled	Death
386	Male	31	Rural	Southwestern	Farmer	Retreatment failure	Not enrolled	Death
387	Male	54	Rural	Central	Farmer	Initial treatment failur	Enrolled	
388	Male	42	Rural	Central	Farmer	New	Not enrolled	Out-migration
389	Male	23	Rural	Northern	Farmer	New	Enrolled	
390	Male	51	Rural	Northern	Farmer	New	Not enrolled	Out-migration
391	Femal	39	Rural	Southern	Farmer	Initial treatment failur	Not enrolled	Out-migration
392	Femal	54	Rural	Northern	Farmer	Relapse	Enrolled	
393	Male	41	Urban	Southern	Unemployed/unreported	Relapse	Enrolled	
394	Male	41	Rural	Northern	Farmer	Relapse	Enrolled	
395	Male	56	Rural	Northern	Farmer	New	Enrolled	
396	Male	42	Urban	Northern	City worker	New	Enrolled	
397	Male	59	Rural	Southwestern	Farmer	Retreatment failure	Enrolled	
398	Male	45	Urban	Central	Unemployed/unreported	New	Enrolled	
399	Male	40	Rural	Southern	Farmer	Relapse	Not enrolled	Out-migration
400	Male	30	Rural	Southern	Farmer	Relapse	Enrolled	
401	Femal	28	Rural	Central	Farmer	Initial treatment failur	Enrolled	
402	Male	59	Rural	Southwestern	Farmer	Retreatment failure	Enrolled	
403	Male	30	Rural	Southern	Farmer	New	Enrolled	
404	Male	55	Rural	Northern	Farmer	Relapse	Enrolled	
405	Male	37	Urban	Northern	City worker	Retreatment failure	Enrolled	
406	Male	57	Rural	Northern	Farmer	Retreatment failure	Enrolled	
407	Male	51	Rural	Northern	Farmer	New	Enrolled	
408	Femal	66	Rural	Northern	Farmer	Relapse	Enrolled	
409	Male	31	Rural	Northern	Farmer	Retreatment failure	Not enrolled	Economic hardship
410	Femal	36	Rural	Northern	Farmer	Retreatment failure	Enrolled	
411	Male	22	Rural	Northern	Farmer	Retreatment failure	Not enrolled	Non-standard treatment
412	Male	60	Urban	Southwestern	City worker	Relapse	Enrolled	
413	Male	40	Urban	Northern	City worker	Retreatment failure	Enrolled	
414	Femal	64	Rural	Central	Farmer	Retreatment failure	Enrolled	
415	Male	48	Rural	Central	Farmer	Relapse	Enrolled	
416	Male	63	Rural	Central	Farmer	New	Enrolled	
417	Male	61	Rural	Southern	Farmer	Retreatment failure	Enrolled	
418	Male	53	Rural	Western	Farmer	Retreatment failure	Enrolled	
419	Male	43	Urban	Southern	Unemployed/unreported	Retreatment failure	Not enrolled	Death
420	Femal	28	Rural	Northern	Farmer	Retreatment failure	Enrolled	
421	Male	19	Urban	Northern	Retired/Student	Retreatment failure	Enrolled	
422	Male	39	Urban	Southern	City worker	Retreatment failure	Enrolled	
423	Male	40	Rural	Central	Farmer	New	Enrolled	
424	Femal	34	Rural	Northern	Farmer	Retreatment failure	Not enrolled	studies

425	Femal	38	Rural	Central	Farmer	Retreatment failure	Enrolled	
426	Male	43	Urban	Northern	City worker	Retreatment failure	Enrolled	
427	Femal	55	Rural	Unknown	Farmer	Smear+ after 3 ms	Not enrolled	Unknown address
428	Male	58	Rural	Northern	Farmer	Relapse	Not enrolled	Economic hardship
429	Femal	35	Rural	Southern	Farmer	Retreatment failure	Enrolled	
430	Femal	48	Rural	Northern	Farmer	Retreatment failure	Enrolled	
431	Femal	18	Urban	Western	Retired/Student	New	Not enrolled	Economic hardship
432	Male	27	Rural	Southern	Farmer	Return or other	Not enrolled	Out-migration
433	Femal	37	Rural	Southern	Farmer	Relapse	Enrolled	
434	Male	47	Urban	Southern	City worker	New	Enrolled	
435	Male	51	Rural	Northern	Farmer	New	Enrolled	
436	Male	55	Urban	Northern	City worker	Retreatment failure	Enrolled	
437	Male	64	Rural	Southwestern	Farmer	New	Not enrolled	Out-migration
438	Femal	35	Rural	Southwestern	Farmer	Retreatment failure	Enrolled	
439	Male	47	Rural	Southern	Farmer	Relapse	Not enrolled	Non-standard treatment
440	Femal	59	Rural	Southwestern	Farmer	New	Not enrolled	Death
441	Femal	33	Urban	Northern	Unemployed/unreported	Relapse	Enrolled	
442	Male	46	Rural	Central	Farmer	Initial treatment failur	Enrolled	
443	Male	27	Rural	Southwestern	Farmer	Relapse	Enrolled	
444	Male	42	Rural	Southern	City worker	Relapse	Enrolled	
445	Male	38	Urban	Northern	City worker	Retreatment failure	Enrolled	
446	Male	41	Urban	Central	City worker	New	Enrolled	
447	Male	40	Rural	Northern	Farmer	Retreatment failure	Enrolled	
448	Male	29	Urban	Southwestern	City worker	Retreatment failure	Enrolled	
449	Male	75	Rural	Southwestern	Farmer	Retreatment failure	Not enrolled	Other severe disease
450	Male	25	Rural	Central	Farmer	New	Not enrolled	Out-migration
451	Femal	24	Urban	Northern	City worker	Retreatment failure	Enrolled	
452	Male	51	Rural	Central	Farmer	Retreatment failure	Enrolled	
453	Male	51	Rural	Central	Farmer	New	Enrolled	
454	Male	41	Rural	Southwestern	Farmer	Retreatment failure	Enrolled	
455	Male	35	Rural	Central	City worker	Retreatment failure	Enrolled	
456	Male	48	Rural	Northern	Farmer	Relapse	Not enrolled	Belief of being cured
457	Male	60	Rural	Southern	Farmer	New	Not enrolled	Economic hardship
458	Male	52	Rural	Western	Farmer	Retreatment failure	Enrolled	
459	Male	58	Urban	Central	City worker	Relapse	Enrolled	
460	Male	46	Rural	Southwestern	Farmer	Relapse	Enrolled	
461	Male	40	Rural	Northern	Farmer	Relapse	Enrolled	
462	Male	52	Rural	Southern	Farmer	Retreatment failure	Enrolled	
463	Male	35	Rural	Northern	Farmer	Retreatment failure	Enrolled	
464	Male	24	Rural	Southwestern	Farmer	Retreatment failure	Enrolled	
465	Male	56	Rural	Northern	Farmer	Retreatment failure	Enrolled	
466	Male	48	Rural	Northern	Farmer	Relapse	Not enrolled	Out-migration
467	Male	32	Rural	Northern	Farmer	Retreatment failure	Enrolled	
468	Male	42	Rural	Southwestern	Farmer	Retreatment failure	Enrolled	
469	Male	29	Rural	Southwestern	Farmer	Retreatment failure	Enrolled	
470	Femal	33	Urban	Northern	Unemployed/unreported	New	Not enrolled	Out-migration
471	Male	46	Rural	Unknown	Farmer	Retreatment failure	Not enrolled	Unknown address
472	Male	52	Rural	Southwestern	Farmer	Retreatment failure	Not enrolled	Economic hardship
473	Male	24	Urban	Northern	City worker	New	Enrolled	
474	Male	41	Rural	Southwestern	Farmer	New	Enrolled	
475	Femal	28	Rural	Southern	Farmer	Retreatment failure	Enrolled	
476	Femal	23	Rural	Southwestern	Farmer	New	Not enrolled	studies
477	Male	47	Rural	Southwestern	Farmer	Relapse	Enrolled	
478	Male	40	Urban	Southwestern	City worker	New	Enrolled	
479	Male	73	Rural	Northern	Farmer	New	Not enrolled	Out-migration
480	Male	30	Urban	Central	City worker	Retreatment failure	Enrolled	
481	Male	44	Urban	Southwestern	City worker	Retreatment failure	Enrolled	
482	Male	33	Rural	Central	Farmer	Return or other	Not enrolled	studies
483	Male	40	Rural	Northern	Farmer	New	Not enrolled	Out-migration
484	Male	71	Rural	Southwestern	Farmer	Retreatment failure	Enrolled	
485	Femal	44	Rural	Central	Farmer	New	Not enrolled	Out-migration

486	Femal	81	Rural	Central	Farmer	Relapse	Not enrolled	Out-migration
487	Male	28	Rural	Southwestern	Farmer	Retreatment failure	Enrolled	
488	Male	21	Rural	Unknown	Farmer	Retreatment failure	Not enrolled	Unknown address
489	Male	56	Rural	Central	Farmer	Retreatment failure	Enrolled	
490	Male	56	Rural	Northern	Farmer	Relapse	Enrolled	
491	Male	70	Rural	Central	Farmer	Retreatment failure	Enrolled	
492	Male	54	Rural	Northern	Farmer	New	Not enrolled	Out-migration
493	Male	62	Rural	Southwestern	Farmer	Relapse	Not enrolled	Out-migration
494	Male	66	Rural	Western	Farmer	Retreatment failure	Not enrolled	Economic hardship
495	Femal	30	Urban	Western	City worker	Retreatment failure	Enrolled	
496	Male	44	Rural	Northern	Farmer	New	Enrolled	
497	Femal	34	Rural	Western	Farmer	Retreatment failure	Enrolled	
498	Male	26	Rural	Southwestern	Farmer	New	Enrolled	
499	Male	22	Rural	Southwestern	Farmer	Relapse	Not enrolled	Non-standard treatment
500	Male	73	Rural	Northern	Farmer	Retreatment failure	Not enrolled	Other severe disease
501	Male	64	Rural	Western	Farmer	Retreatment failure	Enrolled	
502	Male	74	Urban	Central	Retired/Student	Relapse	Enrolled	
503	Femal	54	Rural	Southwestern	Farmer	New	Not enrolled	Out-migration
504	Femal	58	Rural	Southern	Farmer	Initial treatment failur	Enrolled	
505	Femal	49	Rural	Central	Farmer	Retreatment failure	Enrolled	
506	Femal	46	Urban	Southwestern	City worker	Retreatment failure	Enrolled	
507	Femal	40	Rural	Southern	Farmer	Smear+ after 3 ms	Enrolled	
508	Male	36	Rural	Western	Farmer	Retreatment failure	Enrolled	
509	Male	46	Rural	Southwestern	Farmer	New	Not enrolled	Out-migration
510	Male	47	Rural	Northern	Farmer	Retreatment failure	Enrolled	
511	Male	44	Rural	Southern	Farmer	Retreatment failure	Enrolled	
512	Male	43	Urban	Central	City worker	New	Enrolled	
513	Male	78	Rural	Central	Farmer	Retreatment failure	Not enrolled	Out-migration
514	Male	33	Urban	Central	City worker	New	Enrolled	
515	Male	60	Rural	Central	Farmer	New	Not enrolled	Out-migration
516	Femal	48	Rural	Northern	Farmer	Retreatment failure	Not enrolled	studies
517	Male	47	Rural	Southwestern	Farmer	New	Not enrolled	Economic hardship
518	Male	42	Rural	Southern	Farmer	Initial treatment failur	Enrolled	
519	Male	24	Rural	Northern	Farmer	Retreatment failure	Enrolled	
520	Femal	36	Rural	Central	Farmer	Retreatment failure	Enrolled	
521	Male	37	Rural	Western	Farmer	Retreatment failure	Enrolled	
522	Femal	41	Rural	Southern	Farmer	New	Enrolled	
523	Femal	48	Rural	Northern	Farmer	Initial treatment failur	Not enrolled	Concern about work and studies
524	Male	58	Rural	Southern	Farmer	Retreatment failure	Enrolled	
525	Male	47	Urban	Southwestern	City worker	Initial treatment failur	Enrolled	
526	Male	44	Rural	Northern	Farmer	Retreatment failure	Not enrolled	Non-standard treatment
527	Male	74	Rural	Central	Farmer	Retreatment failure	Not enrolled	Other severe disease
528	Male	51	Rural	Southern	Farmer	Retreatment failure	Enrolled	
529	Male	36	Rural	Central	Farmer	New	Not enrolled	Out-migration
530	Male	23	Rural	Central	Farmer	Relapse	Enrolled	
531	Male	50	Rural	Southern	Farmer	New	Not enrolled	Non-standard treatment
532	Male	48	Rural	Central	Farmer	Retreatment failure	Not enrolled	Concern about work and studies
533	Male	33	Rural	Western	Farmer	Initial treatment failur	Enrolled	
534	Femal	26	Urban	Southern	Unemployed/unreported	Relapse	Enrolled	
535	Femal	26	Rural	Southern	Farmer	Relapse	Enrolled	
536	Femal	42	Urban	Southern	Unemployed/unreported	Relapse	Not enrolled	studies
537	Male	57	Rural	Northern	Farmer	Retreatment failure	Enrolled	
538	Male	56	Rural	Northern	Farmer	Retreatment failure	Enrolled	
539	Male	19	Urban	Central	City worker	Relapse	Enrolled	
540	Male	65	Rural	Central	Farmer	Retreatment failure	Enrolled	
541	Male	26	Rural	Central	Farmer	Retreatment failure	Enrolled	
542	Male	45	Rural	Northern	Farmer	Retreatment failure	Not enrolled	Out-migration
543	Femal	24	Rural	Northern	Farmer	Retreatment failure	Not enrolled	Non-standard treatment
544	Male	50	Rural	Southwestern	Farmer	Retreatment failure	Enrolled	
545	Male	22	Rural	Central	Farmer	Retreatment failure	Not enrolled	Non-standard treatment
546	Male	57	Rural	Central	Farmer	Retreatment failure	Enrolled	

547	Femal	29	Rural	Central	Farmer	Retreatment failure	Enrolled	
548	Male	55	Rural	Central	Farmer	Initial treatment failur	Enrolled	
549	Male	55	Rural	Central	Farmer	Relapse	Not enrolled	Economic hardship
550	Femal	24	Rural	Southern	Farmer	Retreatment failure	Enrolled	
551	Femal	48	Rural	Southwestern	Farmer	Relapse	Enrolled	
552	Femal	33	Rural	Central	Farmer	Relapse	Not enrolled	Economic hardship
553	Femal	33	Urban	Central	City worker	Retreatment failure	Enrolled	
554	Male	50	Rural	Northern	Farmer	New	Enrolled	
555	Male	35	Rural	Southwestern	Farmer	Relapse	Enrolled	
556	Femal	43	Rural	Northern	Farmer	New	Not enrolled	Out-migration
557	Male	27	Rural	Southwestern	Farmer	Initial treatment failur	Enrolled	
558	Femal	19	Rural	Southern	Retired/Student	Relapse	Enrolled	
559	Male	50	Rural	Central	Farmer	Retreatment failure	Enrolled	
560	Male	76	Rural	Northern	Farmer	Retreatment failure	Not enrolled	Death
561	Femal	41	Rural	Unknown	Farmer	Retreatment failure	Not enrolled	Unknown address
562	Male	48	Rural	Central	Farmer	Retreatment failure	Not enrolled	Out-migration
563	Male	52	Rural	Central	Farmer	Retreatment failure	Not enrolled	Economic hardship
564	Femal	39	Rural	Central	Farmer	Retreatment failure	Enrolled	
565	Femal	36	Rural	Unknown	Farmer	Initial treatment failur	Not enrolled	Unknown address
566	Male	41	Rural	Unknown	Farmer	Relapse	Not enrolled	Unknown address
567	Femal	35	Rural	Southern	Farmer	Relapse	Enrolled	
568	Male	24	Rural	Southwestern	Farmer	Initial treatment failur	Enrolled	
569	Femal	39	Rural	Southern	Farmer	Retreatment failure	Enrolled	
570	Male	36	Rural	Northern	Farmer	Relapse	Not enrolled	studies
571	Male	44	Urban	Southwestern	City worker	Relapse	Enrolled	
572	Male	62	Rural	Central	Farmer	Relapse	Enrolled	
573	Male	50	Rural	Southern	Farmer	Initial treatment failur	Enrolled	
574	Femal	29	Rural	Northern	Farmer	Relapse	Not enrolled	Non-standard treatment
575	Male	63	Rural	Central	Farmer	New	Not enrolled	Economic hardship
576	Male	37	Urban	Southwestern	Unemployed/unreported	Retreatment failure	Enrolled	
577	Femal	39	Rural	Northern	Farmer	Relapse	Enrolled	
578	Male	56	Rural	Southwestern	Farmer	Smear+ after 3 ms	Enrolled	
579	Male	31	Rural	Unknown	Farmer	Relapse	Not enrolled	Unknown address
580	Male	44	Rural	Southwestern	Farmer	Retreatment failure	Enrolled	
581	Femal	40	Rural	Northern	Farmer	Relapse	Enrolled	
582	Male	44	Rural	Southern	Farmer	Retreatment failure	Enrolled	
583	Male	54	Rural	Southern	Farmer	New	Not enrolled	Death
584	Male	49	Rural	Central	Farmer	Relapse	Not enrolled	Out-migration
585	Male	76	Rural	Southern	Farmer	New	Enrolled	
586	Femal	63	Rural	Central	Farmer	New	Not enrolled	Death
587	Femal	38	Rural	Northern	Farmer	Retreatment failure	Not enrolled	studies
588	Femal	25	Rural	Central	Farmer	Relapse	Not enrolled	Out-migration
589	Male	41	Rural	Central	Farmer	Retreatment failure	Enrolled	
590	Male	59	Rural	Western	Farmer	Retreatment failure	Enrolled	
591	Femal	44	Rural	Northern	Farmer	Retreatment failure	Enrolled	
592	Male	58	Rural	Central	Farmer	Relapse	Enrolled	
593	Male	31	Urban	Southern	City worker	Retreatment failure	Enrolled	
594	Male	60	Rural	Central	Farmer	New	Enrolled	
595	Male	60	Rural	Western	Farmer	New	Not enrolled	Economic hardship
596	Male	39	Rural	Southwestern	Farmer	Retreatment failure	Enrolled	
597	Femal	41	Rural	Southern	Farmer	Return or other	Enrolled	
598	Femal	41	Urban	Southwestern	Unemployed/unreported	Relapse	Not enrolled	Out-migration
599	Male	40	Rural	Northern	Farmer	Retreatment failure	Enrolled	
600	Femal	59	Urban	Southern	City worker	Retreatment failure	Enrolled	
601	Male	38	Rural	Other province	Farmer	Retreatment failure	Enrolled	
602	Male	40	Rural	Southwestern	Farmer	Retreatment failure	Enrolled	
603	Femal	23	Rural	Southern	Farmer	New	Not enrolled	Death
604	Male	32	Rural	Unknown	Farmer	Retreatment failure	Enrolled	
605	Femal	31	Rural	Southwestern	Farmer	Retreatment failure	Not enrolled	Out-migration
606	Male	55	Urban	Southwestern	City worker	Retreatment failure	Enrolled	
607	Femal	51	Rural	Southwestern	Farmer	Retreatment failure	Enrolled	

608	Male	49	Rural	Central	Farmer	Relapse	Enrolled	
609	Male	48	Rural	Southern	Farmer	Initial treatment failur	Not enrolled	Out-migration
610	Male	81	Rural	Central	Farmer	Relapse	Not enrolled	Out-migration
611	Male	73	Rural	Southern	Farmer	Relapse	Enrolled	
612	Male	60	Rural	Southern	Farmer	Retreatment failure	Enrolled	
613	Femal	55	Rural	Southwestern	Farmer	Relapse	Enrolled	
614	Male	34	Urban	Southwestern	City worker	Retreatment failure	Enrolled	
615	Male	49	Rural	Central	Farmer	Retreatment failure	Not enrolled	Economic hardship
616	Male	40	Rural	Southwestern	Farmer	Relapse	Not enrolled	studies
617	Male	33	Urban	Northern	Unemployed/unreported	Initial treatment failur	Not enrolled	studies
618	Male	23	Rural	Northern	Farmer	New	Enrolled	
619	Male	83	Rural	Central	Farmer	Relapse	Not enrolled	Out-migration
620	Male	62	Rural	Southwestern	Farmer	New	Not enrolled	Death
621	Male	48	Rural	Southwestern	Farmer	Relapse	Not enrolled	Out-migration
622	Male	52	Rural	Unknown	Farmer	New	Not enrolled	Unknown address
623	Male	55	Rural	Central	Farmer	Retreatment failure	Enrolled	
624	Male	69	Rural	Southwestern	Farmer	Retreatment failure	Not enrolled	Economic hardship
625	Femal	25	Rural	Northern	Farmer	Relapse	Not enrolled	Out-migration
626	Femal	25	Rural	Northern	Farmer	Retreatment failure	Enrolled	
627	Male	50	Rural	Southwestern	Farmer	Retreatment failure	Enrolled	
628	Male	41	Rural	Southwestern	Farmer	Relapse	Enrolled	
629	Femal	25	Rural	Western	Farmer	Retreatment failure	Enrolled	
630	Male	63	Rural	Northern	Farmer	Retreatment failure	Not enrolled	studies
631	Male	59	Rural	Northern	Farmer	Retreatment failure	Enrolled	
632	Femal	21	Rural	Western	Farmer	Retreatment failure	Enrolled	
633	Male	25	Rural	Southwestern	Farmer	Relapse	Enrolled	
634	Male	33	Rural	Southwestern	Farmer	Retreatment failure	Enrolled	
635	Femal	43	Rural	Western	Farmer	Retreatment failure	Not enrolled	Non-standard treatment
636	Male	45	Rural	Central	Farmer	Initial treatment failur	Not enrolled	Concern about work and studies
637	Male	50	Rural	Northern	Farmer	Initial treatment failur	Not enrolled	Out-migration
638	Femal	32	Rural	Central	Farmer	New	Not enrolled	Belief of being cured
639	Femal	30	Rural	Southern	Farmer	Retreatment failure	Enrolled	
640	Femal	30	Rural	Southwestern	Farmer	Retreatment failure	Enrolled	
641	Male	69	Urban	Central	Retired/Student	Relapse	Not enrolled	Other severe disease
642	Femal	27	Rural	Northern	Farmer	New	Not enrolled	Belief of being cured
643	Male	64	Rural	Northern	Farmer	Relapse	Not enrolled	studies
644	Male	47	Urban	Central	City worker	Initial treatment failur	Enrolled	
645	Male	26	Rural	Northern	Farmer	Retreatment failure	Enrolled	
646	Femal	25	Rural	Central	Farmer	Relapse	Not enrolled	Out-migration
647	Male	39	Rural	Southern	Farmer	Retreatment failure	Enrolled	
648	Femal	26	Rural	Central	Farmer	Retreatment failure	Enrolled	
649	Male	47	Rural	Northern	Farmer	Relapse	Enrolled	
650	Male	20	Rural	Northern	Retired/Student	Initial treatment failur	Enrolled	
651	Male	50	Rural	Southern	Farmer	New	Not enrolled	Non-standard treatment
652	Male	20	Rural	Central	Retired/Student	Relapse	Enrolled	
653	Male	24	Urban	Western	Retired/Student	Relapse	Not enrolled	Concern about work and studies
654	Femal	38	Rural	Southwestern	Farmer	Retreatment failure	Enrolled	
655	Femal	36	Urban	Southwestern	Unemployed/unreported	New	Enrolled	
656	Femal	36	Rural	Southwestern	Farmer	Relapse	Enrolled	
657	Femal	47	Rural	Southwestern	Farmer	Retreatment failure	Enrolled	
658	Male	52	Rural	Southern	Farmer	New	Not enrolled	Economic hardship
659	Male	45	Rural	Central	Farmer	New	Not enrolled	Belief of being cured
660	Male	26	Urban	Southwestern	City worker	Retreatment failure	Enrolled	
661	Femal	49	Rural	Western	Farmer	Retreatment failure	Enrolled	
662	Femal	26	Rural	Southwestern	Farmer	New	Enrolled	
663	Male	48	Rural	Northern	Farmer	Retreatment failure	Enrolled	
664	Male	47	Rural	Central	Farmer	Retreatment failure	Enrolled	
665	Male	48	Rural	Central	City worker	Relapse	Enrolled	
666	Male	57	Urban	Northern	Unemployed/unreported	Retreatment failure	Enrolled	
667	Femal	26	Rural	Southwestern	Farmer	Relapse	Enrolled	
668	Male	54	Rural	Central	Farmer	New	Enrolled	

669	Femal	60	Rural	Central	Farmer	Retreatment failure	Not enrolled	Out-migration
670	Femal	27	Rural	Northern	Farmer	Relapse	Enrolled	
671	Male	28	Rural	Southwestern	Farmer	Retreatment failure	Not enrolled	Out-migration
672	Male	57	Rural	Central	Farmer	Retreatment failure	Enrolled	
673	Male	48	Rural	Southwestern	Farmer	Retreatment failure	Enrolled	
674	Male	54	Rural	Western	Farmer	New	Not enrolled	Death
675	Male	62	Urban	Southwestern	Unemployed/unreported	New	Not enrolled	Economic hardship
676	Male	51	Rural	Central	Farmer	Smear+ after 3 ms	Enrolled	
677	Femal	62	Rural	Southern	Farmer	Relapse	Enrolled	
678	Male	43	Rural	Southwestern	Farmer	Relapse	Enrolled	
679	Male	28	Rural	Northern	Farmer	New	Not enrolled	studies
680	Male	37	Rural	Southern	Farmer	Retreatment failure	Enrolled	
681	Male	42	Rural	Southwestern	Farmer	Retreatment failure	Enrolled	
682	Femal	21	Rural	Central	Farmer	Retreatment failure	Not enrolled	studies
683	Male	51	Urban	Northern	Unemployed/unreported	Retreatment failure	Enrolled	
684	Male	41	Rural	Northern	Farmer	Relapse	Not enrolled	Economic hardship
685	Male	45	Urban	Southwestern	Unemployed/unreported	Relapse	Enrolled	
686	Male	85	Rural	Central	Farmer	Retreatment failure	Enrolled	
687	Male	57	Rural	Northern	Farmer	Retreatment failure	Enrolled	
688	Male	45	Rural	Northern	Farmer	Initial treatment failur	Enrolled	
689	Femal	63	Rural	Northern	Farmer	Relapse	Enrolled	
690	Femal	65	Rural	Central	Farmer	Retreatment failure	Enrolled	
691	Male	46	Urban	Southwestern	Unemployed/unreported	Retreatment failure	Not enrolled	Economic hardship
692	Male	43	Urban	Southwestern	City worker	Retreatment failure	Enrolled	
693	Femal	67	Rural	Northern	Farmer	Retreatment failure	Enrolled	
694	Femal	27	Rural	Southwestern	Farmer	Relapse	Not enrolled	Out-migration
695	Male	72	Urban	Northern	Retired/Student	Relapse	Enrolled	
696	Femal	28	Rural	Central	Farmer	New	Not enrolled	Out-migration
697	Femal	30	Rural	Northern	Farmer	Retreatment failure	Not enrolled	Out-migration
698	Male	38	Rural	Southwestern	Farmer	Retreatment failure	Enrolled	
699	Male	65	Rural	Northern	Farmer	Retreatment failure	Enrolled	
700	Femal	43	Urban	Southwestern	Unemployed/unreported	Relapse	Not enrolled	Out-migration
701	Femal	75	Rural	Southwestern	Farmer	New	Not enrolled	Economic hardship
702	Femal	28	Rural	Southwestern	Farmer	Retreatment failure	Enrolled	
703	Femal	82	Rural	Southern	Farmer	Relapse	Not enrolled	Economic hardship
704	Male	59	Rural	Southern	Farmer	Relapse	Enrolled	
705	Male	47	Rural	Northern	Farmer	Retreatment failure	Enrolled	
706	Male	26	Urban	Southwestern	Unemployed/unreported	Retreatment failure	Enrolled	
707	Male	37	Rural	Southwestern	Farmer	Relapse	Enrolled	
708	Femal	39	Rural	Southern	Farmer	Relapse	Enrolled	
709	Femal	50	Rural	Southern	Farmer	Retreatment failure	Not enrolled	Belief of being cured
710	Femal	43	Urban	Southwestern	City worker	Retreatment failure	Enrolled	
711	Male	29	Rural	Southwestern	Farmer	Retreatment failure	Enrolled	
712	Male	50	Rural	Southern	Farmer	Retreatment failure	Enrolled	
713	Male	46	Urban	Southern	Unemployed/unreported	Retreatment failure	Enrolled	
714	Male	22	Rural	Southwestern	Farmer	Retreatment failure	Enrolled	
715	Male	40	Rural	Western	Farmer	New	Not enrolled	Out-migration
716	Male	42	Rural	Southwestern	Farmer	Retreatment failure	Enrolled	
717	Male	33	Rural	Northern	Farmer	New	Enrolled	
718	Male	49	Urban	Northern	City worker	Retreatment failure	Enrolled	
719	Male	65	Rural	Southwestern	Farmer	Retreatment failure	Not enrolled	Economic hardship
720	Femal	32	Rural	Southwestern	Farmer	Retreatment failure	Enrolled	
721	Male	54	Rural	Southern	Farmer	Retreatment failure	Enrolled	
722	Male	41	Rural	Southwestern	Farmer	Retreatment failure	Enrolled	
723	Male	23	Rural	Southern	Farmer	Relapse	Enrolled	
724	Femal	76	Rural	Northern	Farmer	Retreatment failure	Not enrolled	Other severe disease
725	Male	66	Rural	Central	Farmer	Relapse	Not enrolled	Economic hardship
726	Male	47	Rural	Southwestern	Farmer	Initial treatment failur	Enrolled	
727	Male	27	Rural	Southern	Farmer	Retreatment failure	Enrolled	
728	Male	36	Rural	Unknown	Farmer	New	Not enrolled	Unknown address
729	Femal	44	Rural	Northern	Farmer	Relapse	Enrolled	

730	Male	31	Rural	Northern	Farmer	Relapse	Not enrolled	Out-migration
731	Male	53	Rural	Southwestern	Farmer	Relapse	Not enrolled	Economic hardship
732	Male	43	Rural	Northern	Farmer	Retreatment failure	Enrolled	
733	Male	69	Rural	Southwestern	Farmer	Relapse	Not enrolled	Economic hardship
734	Femal	43	Rural	Southwestern	Farmer	Relapse	Enrolled	
735	Male	49	Urban	Northern	Unemployed/unreported	Relapse	Enrolled	
736	Male	20	Rural	Northern	City worker	Retreatment failure	Enrolled	
737	Male	31	Rural	Central	Farmer	Initial treatment failur	Enrolled	
738	Male	33	Urban	Central	Unemployed/unreported	Retreatment failure	Not enrolled	Non-standard treatment
739	Male	33	Urban	Southwestern	Unemployed/unreported	New	Not enrolled	Non-standard treatment
740	Male	27	Rural	Southwestern	Farmer	New	Not enrolled	Out-migration
741	Male	34	Rural	Southwestern	Farmer	Retreatment failure	Enrolled	
742	Male	40	Rural	Central	Farmer	Relapse	Not enrolled	Out-migration
743	Male	42	Urban	Northern	City worker	Retreatment failure	Enrolled	
744	Male	65	Rural	Northern	Farmer	Relapse	Not enrolled	Out-migration
745	Male	28	Rural	Northern	Farmer	Retreatment failure	Enrolled	
746	Male	21	Rural	Southwestern	Farmer	New	Enrolled	
747	Male	59	Urban	Central	City worker	New	Enrolled	
748	Male	49	Rural	Southwestern	Farmer	Retreatment failure	Not enrolled	Economic hardship
749	Femal	32	Rural	Central	Farmer	Retreatment failure	Enrolled	
750	Male	42	Rural	Southern	Farmer	Retreatment failure	Enrolled	
751	Femal	44	Rural	Unknown	Farmer	Retreatment failure	Enrolled	
752	Femal	22	Urban	Northern	City worker	Retreatment failure	Enrolled	
753	Male	32	Rural	Southwestern	Farmer	Retreatment failure	Not enrolled	Belief of being cured
754	Male	54	Rural	Southern	Farmer	Retreatment failure	Enrolled	
755	Male	58	Rural	Northern	Farmer	Retreatment failure	Not enrolled	Out-migration
756	Male	61	Rural	Central	Farmer	Relapse	Not enrolled	Non-standard treatment
757	Male	83	Rural	Northern	Farmer	Initial treatment failur	Not enrolled	Death
758	Male	82	Rural	Northern	Farmer	Retreatment failure	Enrolled	
759	Male	50	Rural	Southwestern	Farmer	Retreatment failure	Enrolled	
760	Male	51	Rural	Southwestern	Farmer	Retreatment failure	Enrolled	
761	Male	46	Rural	Northern	Farmer	Relapse	Not enrolled	Concern about work and studies
762	Male	49	Urban	Northern	City worker	Retreatment failure	Enrolled	
763	Male	55	Urban	Central	City worker	Retreatment failure	Enrolled	
764	Male	47	Rural	Central	Farmer	Retreatment failure	Enrolled	
765	Male	24	Rural	Southwestern	Farmer	Retreatment failure	Not enrolled	Non-standard treatment
766	Femal	76	Rural	Central	Farmer	New	Enrolled	
767	Male	54	Rural	Northern	Farmer	Retreatment failure	Enrolled	
768	Femal	49	Rural	Northern	Farmer	Initial treatment failur	Not enrolled	Out-migration
769	Femal	55	Rural	Southern	Farmer	Retreatment failure	Enrolled	
770	Male	65	Rural	Northern	Farmer	Retreatment failure	Enrolled	
771	Male	22	Rural	Southern	Farmer	New	Enrolled	
772	Male	61	Urban	Southwestern	Retired/Student	New	Not enrolled	Economic hardship
773	Male	64	Rural	Southwestern	Farmer	Relapse	Enrolled	
774	Male	45	Rural	Southern	Farmer	Retreatment failure	Enrolled	
775	Male	60	Rural	Southwestern	Farmer	Relapse	Not enrolled	studies
776	Male	27	Rural	Western	Farmer	Relapse	Enrolled	
777	Femal	24	Rural	Central	Farmer	Relapse	Not enrolled	Out-migration
778	Male	63	Rural	Southern	Farmer	Retreatment failure	Enrolled	
779	Male	41	Urban	Southern	City worker	New	Enrolled	
780	Male	29	Rural	Southwestern	Farmer	Retreatment failure	Not enrolled	Out-migration
781	Male	39	Rural	Western	Farmer	Retreatment failure	Not enrolled	Death
782	Male	31	Rural	Northern	Farmer	Retreatment failure	Enrolled	
783	Male	47	Rural	Northern	Farmer	Retreatment failure	Enrolled	
784	Male	47	Rural	Northern	Farmer	Retreatment failure	Enrolled	
785	Male	46	Rural	Northern	Farmer	New	Not enrolled	Out-migration
786	Femal	25	Rural	Central	Farmer	Retreatment failure	Enrolled	
787	Male	25	Urban	Southern	Unemployed/unreported	Retreatment failure	Enrolled	
788	Male	48	Rural	Northern	Farmer	Retreatment failure	Enrolled	
789	Femal	20	Rural	Southwestern	Retired/Student	Return or other	Enrolled	
790	Male	40	Rural	Southwestern	Farmer	Retreatment failure	Not enrolled	Concern about work and studies

791	Male	37	Rural	Western	Farmer	Retreatment failure	Not enrolled	Belief of being cured
792	Male	48	Rural	Central	Farmer	Retreatment failure	Enrolled	
793	Male	62	Rural	Northern	Farmer	New	Not enrolled	Economic hardship
794	Male	54	Rural	Northern	Farmer	Retreatment failure	Enrolled	
795	Femal	42	Rural	Central	Farmer	New	Enrolled	
796	Male	32	Rural	Central	Farmer	Relapse	Enrolled	
797	Male	26	Rural	Southern	Farmer	Retreatment failure	Enrolled	
798	Male	32	Rural	Northern	Farmer	Retreatment failure	Enrolled	
799	Femal	55	Rural	Southwestern	Farmer	Retreatment failure	Enrolled	
800	Male	52	Rural	Northern	Farmer	Retreatment failure	Enrolled	
801	Male	25	Rural	Western	Farmer	New	Enrolled	
802	Male	44	Urban	Southwestern	City worker	Retreatment failure	Enrolled	
803	Male	25	Rural	Western	Farmer	Retreatment failure	Enrolled	
804	Male	59	Rural	Southwestern	Farmer	Relapse	Enrolled	
805	Male	47	Urban	Northern	Unemployed/unreported	Retreatment failure	Not enrolled	studies
806	Femal	43	Rural	Western	Farmer	Relapse	Enrolled	
807	Male	48	Rural	Western	Farmer	Retreatment failure	Not enrolled	Concern about work and studies
808	Male	38	Rural	Northern	Farmer	New	Enrolled	
809	Male	48	Rural	Northern	Farmer	Retreatment failure	Enrolled	
810	Male	50	Rural	Central	Farmer	Initial treatment failur	Not enrolled	Economic hardship
811	Male	26	Rural	Central	Farmer	Relapse	Enrolled	
812	Male	32	Rural	Southwestern	Farmer	Relapse	Enrolled	
813	Femal	24	Rural	Northern	Farmer	Retreatment failure	Enrolled	
814	Femal	35	Rural	Southern	Farmer	Retreatment failure	Enrolled	
815	Male	49	Rural	Central	Farmer	Initial treatment failur	Enrolled	
816	Male	33	Rural	Northern	Farmer	Retreatment failure	Not enrolled	Death
817	Femal	27	Urban	Central	City worker	Retreatment failure	Enrolled	
818	Male	29	Urban	Southern	City worker	Relapse	Enrolled	
819	Male	52	Rural	Northern	Farmer	Relapse	Enrolled	
820	Femal	46	Rural	Southwestern	Farmer	Relapse	Not enrolled	Concern about work and studies
821	Male	64	Urban	Southwestern	Retired/Student	Retreatment failure	Enrolled	
822	Male	39	Rural	Central	Farmer	Retreatment failure	Enrolled	
823	Male	48	Rural	Southwestern	Farmer	Retreatment failure	Enrolled	
824	Male	59	Rural	Northern	Farmer	Retreatment failure	Enrolled	
825	Femal	29	Rural	Northern	Farmer	Retreatment failure	Enrolled	
826	Femal	34	Urban	Southern	City worker	Retreatment failure	Enrolled	
827	Femal	43	Rural	Southwestern	Farmer	Smear+ after 3 ms	Enrolled	
828	Male	67	Urban	Southwestern	City worker	Retreatment failure	Enrolled	
829	Male	48	Rural	Southwestern	Farmer	New	Enrolled	
830	Male	59	Rural	Southern	Farmer	Retreatment failure	Enrolled	
831	Male	42	Rural	Northern	Farmer	New	Not enrolled	Out-migration
832	Male	67	Rural	Central	Farmer	New	Not enrolled	Economic hardship
833	Male	17	Rural	Southern	Farmer	Relapse	Not enrolled	Out-migration
834	Male	37	Urban	Northern	City worker	Retreatment failure	Enrolled	
835	Male	22	Urban	Southwestern	City worker	Retreatment failure	Enrolled	
836	Male	74	Rural	Southwestern	Farmer	Relapse	Enrolled	
837	Male	50	Rural	Northern	Farmer	Retreatment failure	Not enrolled	Death
838	Femal	43	Rural	Northern	City worker	Retreatment failure	Enrolled	
839	Femal	67	Rural	Southwestern	Farmer	Retreatment failure	Enrolled	
840	Male	57	Rural	Northern	Farmer	Retreatment failure	Enrolled	
841	Male	55	Urban	Southwestern	Unemployed/unreported	Retreatment failure	Not enrolled	Non-standard treatment
842	Male	59	Rural	Southern	Farmer	New	Enrolled	
843	Femal	33	Urban	Southern	Unemployed/unreported	Retreatment failure	Enrolled	
844	Femal	38	Rural	Northern	Farmer	Initial treatment failur	Enrolled	
845	Male	27	Rural	Unknown	Farmer	Relapse	Not enrolled	Unknown address
846	Male	27	Rural	Central	Farmer	New	Enrolled	
847	Male	41	Rural	Southern	Farmer	Relapse	Not enrolled	Out-migration
848	Femal	54	Rural	Central	Farmer	Initial treatment failur	Not enrolled	Economic hardship
849	Male	29	Rural	Southwestern	Farmer	Retreatment failure	Enrolled	
850	Male	49	Urban	Southern	City worker	New	Enrolled	
851	Femal	44	Urban	Northern	City worker	Retreatment failure	Enrolled	

852	Male	66	Rural	Southwestern	Farmer	Retreatment failure	Not enrolled	Death
853	Femal	26	Rural	Central	Farmer	Retreatment failure	Enrolled	
854	Femal	54	Rural	Central	Farmer	Retreatment failure	Enrolled	
855	Male	38	Rural	Southern	Farmer	Relapse	Enrolled	
856	Male	32	Urban	Southern	City worker	Relapse	Enrolled	
857	Femal	20	Rural	Northern	Farmer	New	Enrolled	
858	Male	69	Rural	Central	Farmer	Relapse	Not enrolled	Death
859	Male	71	Rural	Northern	Farmer	New	Not enrolled	Non-standard treatment
860	Male	54	Rural	Central	Farmer	New	Enrolled	
861	Male	41	Urban	Central	Unemployed/unreported	New	Not enrolled	Out-migration
862	Male	49	Rural	Central	Farmer	Retreatment failure	Enrolled	
863	Male	43	Rural	Southwestern	Farmer	Initial treatment failur	Not enrolled	Belief of being cured
864	Male	26	Rural	Northern	Farmer	Relapse	Not enrolled	Out-migration
865	Male	72	Urban	Central	Retired/Student	New	Enrolled	
866	Male	77	Rural	Central	Farmer	Retreatment failure	Enrolled	
867	Male	60	Rural	Southern	Farmer	Retreatment failure	Not enrolled	Non-standard treatment
868	Male	46	Urban	Southern	City worker	Retreatment failure	Enrolled	
869	Male	68	Rural	Northern	Farmer	New	Enrolled	
870	Femal	53	Rural	Southern	Farmer	Retreatment failure	Not enrolled	Death
871	Male	62	Rural	Southern	Farmer	Retreatment failure	Not enrolled	Economic hardship
872	Femal	39	Rural	Southern	City worker	Relapse	Enrolled	
873	Femal	46	Rural	Northern	Farmer	Retreatment failure	Enrolled	
874	Femal	20	Urban	Western	Unemployed/unreported	Relapse	Not enrolled	Belief of being cured
875	Male	27	Rural	Southwestern	Farmer	Retreatment failure	Enrolled	
876	Male	28	Urban	Other province	City worker	Retreatment failure	Enrolled	
877	Male	73	Rural	Northern	Farmer	New	Not enrolled	Belief of being cured
878	Femal	54	Rural	Central	Farmer	Relapse	Not enrolled	Belief of being cured
879	Femal	38	Rural	Southern	Farmer	Retreatment failure	Enrolled	
880	Male	46	Rural	Southwestern	Farmer	Retreatment failure	Enrolled	
881	Male	30	Rural	Northern	Farmer	New	Enrolled	
882	Male	57	Rural	Southwestern	Farmer	Retreatment failure	Not enrolled	Death
883	Femal	58	Rural	Southern	Farmer	Retreatment failure	Not enrolled	Death
884	Male	47	Rural	Southern	Farmer	New	Not enrolled	Non-standard treatment
885	Male	46	Urban	Southern	City worker	Relapse	Enrolled	
886	Male	59	Urban	Southern	Unemployed/unreported	Relapse	Enrolled	
887	Femal	22	Rural	Central	Farmer	New	Enrolled	
888	Male	39	Urban	Northern	City worker	Retreatment failure	Enrolled	
889	Male	58	Urban	Southern	City worker	New	Not enrolled	Concern about work and studies
890	Male	32	Rural	Southwestern	Farmer	Relapse	Enrolled	
891	Male	48	Rural	Southwestern	Farmer	Retreatment failure	Enrolled	
892	Male	43	Rural	Southern	Farmer	Retreatment failure	Enrolled	
893	Male	30	Rural	Central	Farmer	Retreatment failure	Enrolled	
894	Male	60	Rural	Southwestern	Farmer	Relapse	Not enrolled	Belief of being cured
895	Femal	31	Rural	Central	Farmer	Retreatment failure	Enrolled	
896	Femal	53	Rural	Southern	Farmer	Relapse	Enrolled	
897	Male	22	Rural	Southwestern	Farmer	New	Enrolled	
898	Femal	20	Rural	Western	Farmer	New	Enrolled	
899	Femal	47	Rural	Western	Farmer	Retreatment failure	Enrolled	
900	Male	49	Urban	Central	City worker	Retreatment failure	Enrolled	
901	Male	41	Rural	Unknown	Farmer	Relapse	Not enrolled	Unknown address
902	Male	31	Urban	Southwestern	Unemployed/unreported	Retreatment failure	Not enrolled	studies
903	Male	43	Rural	Western	Farmer	Retreatment failure	Enrolled	
904	Male	51	Urban	Southern	City worker	Initial treatment failur	Enrolled	
905	Male	46	Urban	Northern	City worker	Retreatment failure	Enrolled	
906	Femal	37	Urban	Western	City worker	Retreatment failure	Enrolled	
907	Male	55	Rural	Northern	City worker	Retreatment failure	Enrolled	
908	Male	57	Urban	Northern	Unemployed/unreported	Retreatment failure	Enrolled	
909	Femal	17	Rural	Central	Retired/Student	Retreatment failure	Enrolled	
910	Femal	52	Rural	Central	Farmer	Retreatment failure	Enrolled	
911	Male	64	Rural	Central	Farmer	Retreatment failure	Not enrolled	Economic hardship
912	Male	40	Urban	Northern	Unemployed/unreported	Retreatment failure	Enrolled	

913	Male	58	Urban	Northern	City worker	Relapse	Enrolled	
914	Male	37	Rural	Southern	Farmer	Relapse	Enrolled	
915	Male	57	Rural	Northern	City worker	Retreatment failure	Enrolled	
916	Male	42	Rural	Southern	Farmer	New	Not enrolled	studies
917	Femal	26	Rural	Northern	Farmer	Relapse	Enrolled	
918	Male	46	Urban	Central	City worker	Retreatment failure	Enrolled	
919	Male	62	Urban	Southern	Retired/Student	Relapse	Enrolled	
920	Femal	45	Urban	Southern	City worker	Retreatment failure	Enrolled	
921	Femal	36	Rural	Southwestern	Farmer	Retreatment failure	Not enrolled	Belief of being cured
922	Femal	50	Rural	Central	Farmer	Relapse	Enrolled	
923	Male	65	Rural	Northern	Farmer	New	Enrolled	
924	Male	24	Rural	Northern	Farmer	Initial treatment failur	Enrolled	
925	Femal	35	Rural	Southwestern	Farmer	Retreatment failure	Enrolled	
926	Male	24	Rural	Northern	Farmer	Return or other	Enrolled	
927	Male	50	Urban	Central	City worker	New	Enrolled	
928	Male	37	Rural	Northern	Farmer	New	Not enrolled	Out-migration
929	Male	48	Rural	Central	Farmer	Relapse	Not enrolled	Concern about work and studies
930	Male	46	Urban	Southwestern	City worker	Retreatment failure	Enrolled	
931	Male	55	Urban	Southwestern	Unemployed/unreported	New	Not enrolled	Death
932	Femal	18	Rural	Western	Farmer	Retreatment failure	Not enrolled	Belief of being cured
933	Male	48	Urban	Northern	City worker	New	Enrolled	
934	Femal	43	Urban	Southern	City worker	Retreatment failure	Enrolled	
935	Male	14	Urban	Northern	Retired/Student	Relapse	Not enrolled	Non-standard treatment
936	Male	72	Rural	Central	Farmer	New	Not enrolled	Belief of being cured
937	Male	19	Rural	Northern	Retired/Student	Relapse	Enrolled	
938	Male	27	Rural	Unknown	Farmer	Retreatment failure	Not enrolled	Unknown address
939	Male	41	Urban	Southwestern	City worker	Retreatment failure	Enrolled	
940	Femal	25	Rural	Northern	Farmer	Relapse	Enrolled	
941	Male	68	Rural	Southwestern	Farmer	Retreatment failure	Not enrolled	Non-standard treatment
942	Male	61	Rural	Southern	Farmer	Retreatment failure	Enrolled	
943	Male	24	Rural	Northern	Farmer	Retreatment failure	Enrolled	
944	Male	68	Rural	Northern	Farmer	New	Not enrolled	Belief of being cured
945	Male	48	Urban	Southern	Unemployed/unreported	Retreatment failure	Enrolled	
946	Male	61	Urban	Central	Retired/Student	Retreatment failure	Enrolled	
947	Femal	52	Urban	Southern	Unemployed/unreported	Initial treatment failur	Enrolled	
948	Femal	53	Rural	Central	Farmer	New	Not enrolled	Belief of being cured
949	Male	45	Urban	Northern	City worker	Retreatment failure	Enrolled	
950	Femal	52	Urban	Northern	City worker	New	Enrolled	
951	Male	18	Rural	Northern	City worker	Initial treatment failur	Enrolled	
952	Femal	27	Rural	Central	Farmer	Retreatment failure	Not enrolled	Other severe disease
953	Male	18	Rural	Northern	Retired/Student	New	Enrolled	
954	Male	59	Urban	Southwestern	City worker	Initial treatment failur	Enrolled	
955	Male	20	Rural	Central	Farmer	Initial treatment failur	Enrolled	
956	Male	54	Rural	Northern	Farmer	Smear+ after 3 ms	Not enrolled	Death
957	Male	31	Urban	Southern	City worker	New	Enrolled	
958	Femal	31	Rural	Southern	Farmer	Initial treatment failur	Not enrolled	Belief of being cured
959	Male	61	Rural	Southwestern	Farmer	Relapse	Not enrolled	Economic hardship
960	Male	49	Rural	Western	Farmer	New	Enrolled	
961	Femal	18	Urban	Central	Retired/Student	Retreatment failure	Enrolled	
962	Male	35	Urban	Western	Unemployed/unreported	Relapse	Enrolled	
963	Male	19	Rural	Southern	Retired/Student	Retreatment failure	Enrolled	
964	Male	35	Rural	Southern	Farmer	Retreatment failure	Enrolled	
965	Male	65	Urban	Southern	Retired/Student	New	Enrolled	
966	Male	60	Urban	Northern	Unemployed/unreported	Retreatment failure	Enrolled	
967	Male	50	Rural	Unknown	Farmer	Initial treatment failur	Not enrolled	Unknown address
968	Femal	39	Rural	Northern	Farmer	Retreatment failure	Enrolled	
969	Male	58	Rural	Central	Farmer	New	Enrolled	
970	Male	55	Rural	Central	Farmer	Retreatment failure	Enrolled	
971	Male	51	Rural	Central	Farmer	Initial treatment failur	Enrolled	
972	Male	43	Rural	Northern	Farmer	Relapse	Not enrolled	Out-migration
973	Male	61	Rural	Northern	Farmer	Relapse	Not enrolled	Belief of being cured

974	Male	49	Urban	Southern	Unemployed/unreported	New	Enrolled	
975	Femal	44	Rural	Southwestern	Farmer	Relapse	Enrolled	
976	Male	49	Rural	Unknown	Farmer	Smear+ after 3 ms	Not enrolled	Unknown address
977	Male	48	Urban	Central	City worker	Retreatment failure	Enrolled	
978	Femal	46	Rural	Southwestern	Farmer	Relapse	Enrolled	
979	Male	29	Urban	Western	City worker	Relapse	Enrolled	
980	Male	39	Rural	Southwestern	Farmer	New	Enrolled	
981	Male	73	Rural	Central	Farmer	Smear+ after 3 ms	Enrolled	
982	Male	27	Rural	Other province	Farmer	New	Not enrolled	Migrants from other provinces
983	Male	44	Rural	Unknown	Farmer	Retreatment failure	Not enrolled	Unknown address
984	Male	43	Rural	Southwestern	Farmer	Relapse	Not enrolled	Belief of being cured
985	Male	45	Rural	Northern	Farmer	Relapse	Not enrolled	Death
986	Male	49	Rural	Central	Farmer	New	Enrolled	
987	Male	26	Urban	Central	Unemployed/unreported	Relapse	Not enrolled	studies
988	Femal	34	Rural	Central	Farmer	Retreatment failure	Not enrolled	Belief of being cured
989	Femal	31	Rural	Northern	Farmer	Retreatment failure	Enrolled	
990	Male	57	Rural	Central	Farmer	Relapse	Enrolled	
991	Male	37	Rural	Western	Farmer	Retreatment failure	Not enrolled	Other severe disease
992	Male	57	Rural	Central	Farmer	Retreatment failure	Enrolled	
993	Male	55	Rural	Southern	Farmer	Initial treatment failur	Enrolled	
994	Femal	30	Urban	Western	City worker	Relapse	Enrolled	
995	Male	28	Rural	Central	Farmer	Retreatment failure	Enrolled	
996	Male	63	Rural	Northern	Farmer	Retreatment failure	Not enrolled	Economic hardship
997	Male	57	Rural	Central	Farmer	Initial treatment failur	Enrolled	
998	Male	40	Rural	Southwestern	City worker	Relapse	Enrolled	
999	Femal	60	Rural	Northern	Farmer	Relapse	Enrolled	
1000	Male	54	Rural	Southern	Farmer	Retreatment failure	Enrolled	
1001	Male	70	Rural	Northern	Retired/Student	Relapse	Enrolled	
1002	Male	57	Urban	Other province	Unemployed/unreported	Retreatment failure	Enrolled	
1003	Male	23	Rural	Northern	Farmer	Relapse	Enrolled	
1004	Male	57	Urban	Northern	City worker	Retreatment failure	Enrolled	
1005	Male	67	Rural	Central	Farmer	New	Not enrolled	Other severe disease
1006	Femal	43	Rural	Western	Farmer	Retreatment failure	Not enrolled	Economic hardship
1007	Male	74	Urban	Central	Retired/Student	Relapse	Enrolled	
1008	Male	46	Rural	Other province	Farmer	New	Not enrolled	Migrants from other provinces
1009	Male	47	Rural	Central	Farmer	Relapse	Not enrolled	Belief of being cured
1010	Male	50	Rural	Southern	Farmer	New	Enrolled	
1011	Male	48	Urban	Western	Unemployed/unreported	New	Enrolled	
1012	Male	48	Rural	Southern	Farmer	Relapse	Enrolled	
1013	Male	30	Urban	Southwestern	Unemployed/unreported	New	Enrolled	
1014	Male	50	Rural	Southwestern	Farmer	New	Enrolled	
1015	Male	56	Urban	Southwestern	Unemployed/unreported	New	Enrolled	
1016	Male	51	Urban	Southern	City worker	New	Not enrolled	studies
1017	Femal	26	Rural	Northern	Farmer	New	Enrolled	
1018	Male	75	Rural	Northern	Farmer	Retreatment failure	Enrolled	
1019	Femal	31	Rural	Southwestern	Farmer	New	Not enrolled	Other severe disease
1020	Femal	50	Rural	Northern	Farmer	Relapse	Not enrolled	Economic hardship
1021	Femal	37	Rural	Unknown	Farmer	Retreatment failure	Not enrolled	Unknown address
1022	Male	42	Urban	Northern	Unemployed/unreported	Retreatment failure	Enrolled	
1023	Male	33	Rural	Southwestern	Farmer	Relapse	Not enrolled	Belief of being cured
1024	Male	34	Rural	Western	Farmer	New	Enrolled	
1025	Male	34	Rural	Southwestern	Farmer	New	Enrolled	
1026	Femal	23	Urban	Southwestern	City worker	Retreatment failure	Enrolled	
1027	Femal	28	Rural	Southwestern	Farmer	Initial treatment failur	Enrolled	
1028	Femal	49	Urban	Southwestern	Unemployed/unreported	Retreatment failure	Enrolled	
1029	Femal	16	Rural	Western	Retired/Student	Relapse	Enrolled	
1030	Femal	44	Urban	Northern	City worker	Smear+ after 3 ms	Enrolled	
1031	Femal	44	Rural	Northern	Farmer	Relapse	Not enrolled	Death
1032	Male	46	Rural	Northern	Farmer	Retreatment failure	Not enrolled	Concern about work and studies
1033	Male	40	Rural	Western	Farmer	Retreatment failure	Enrolled	
1034	Male	81	Rural	Southwestern	Retired/Student	Retreatment failure	Enrolled	

1035	Male	45	Rural	Northern	Farmer	New	Enrolled	
1036	Femal	41	Rural	Northern	Farmer	Return or other	Enrolled	
1037	Femal	51	Rural	Southwestern	Farmer	Retreatment failure	Enrolled	
1038	Male	57	Urban	Southwestern	Unemployed/unreported	New	Enrolled	
1039	Femal	40	Urban	Northern	Unemployed/unreported	Relapse	Not enrolled	Belief of being cured
1040	Male	47	Rural	Southwestern	Farmer	Retreatment failure	Enrolled	
1041	Femal	65	Urban	Central	Retired/Student	Retreatment failure	Not enrolled	Out-migration
1042	Male	48	Rural	Northern	Farmer	Relapse	Not enrolled	Economic hardship
1043	Femal	49	Urban	Northern	Unemployed/unreported	Relapse	Enrolled	
1044	Femal	25	Rural	Central	Farmer	New	Enrolled	
1045	Male	36	Rural	Southwestern	Farmer	Relapse	Enrolled	
1046	Femal	33	Urban	Southwestern	City worker	Return or other	Enrolled	
1047	Femal	23	Rural	Southwestern	Farmer	New	Enrolled	
1048	Male	53	Urban	Southern	City worker	Retreatment failure	Enrolled	
1049	Femal	61	Rural	Northern	Farmer	New	Not enrolled	Death
1050	Femal	39	Urban	Unknown	Unemployed/unreported	Retreatment failure	Enrolled	
1051	Male	32	Rural	Central	Farmer	New	Enrolled	
1052	Male	22	Rural	Northern	Farmer	Relapse	Not enrolled	Non-standard treatment
1053	Male	50	Urban	Central	City worker	Relapse	Enrolled	
1054	Femal	24	Urban	Central	Unemployed/unreported	New	Enrolled	
1055	Male	41	Rural	Southwestern	Farmer	New	Enrolled	
1056	Male	38	Rural	Southern	Farmer	Relapse	Enrolled	
1057	Femal	30	Urban	Southwestern	City worker	Retreatment failure	Enrolled	
1058	Femal	39	Rural	Northern	Farmer	Relapse	Not enrolled	Non-standard treatment
1059	Male	53	Rural	Central	Farmer	Initial treatment failur	Not enrolled	Economic hardship
1060	Male	49	Rural	Southwestern	Farmer	Retreatment failure	Enrolled	
1061	Femal	24	Rural	Southern	Farmer	New	Enrolled	
1062	Male	60	Rural	Southwestern	Farmer	Initial treatment failur	Not enrolled	Death
1063	Male	35	Rural	Southern	City worker	Retreatment failure	Enrolled	
1064	Male	50	Urban	Southern	City worker	New	Enrolled	
1065	Male	63	Urban	Southwestern	Retired/Student	Retreatment failure	Enrolled	
1066	Femal	24	Rural	Southwestern	Farmer	Retreatment failure	Enrolled	
1067	Male	22	Urban	Southwestern	City worker	New	Enrolled	
1068	Male	22	Urban	Western	Unemployed/unreported	Relapse	Not enrolled	Belief of being cured
1069	Male	52	Rural	Central	Farmer	Relapse	Not enrolled	Economic hardship
1070	Male	25	Urban	Southwestern	Unemployed/unreported	Relapse	Enrolled	
1071	Male	27	Rural	Southern	Farmer	Retreatment failure	Enrolled	
1072	Male	30	Rural	Southwestern	Farmer	Relapse	Not enrolled	Belief of being cured
1073	Femal	56	Rural	Southwestern	Farmer	New	Not enrolled	Economic hardship
1074	Male	53	Rural	Central	Farmer	Retreatment failure	Enrolled	
1075	Femal	58	Rural	Central	Farmer	New	Not enrolled	Non-standard treatment
1076	Male	28	Rural	Northern	Farmer	Initial treatment failur	Enrolled	
1077	Femal	35	Rural	Northern	Farmer	Relapse	Not enrolled	Out-migration
1078	Femal	23	Rural	Northern	Farmer	New	Enrolled	
1079	Male	17	Rural	Central	Retired/Student	Retreatment failure	Enrolled	
1080	Male	40	Rural	Southwestern	Farmer	New	Not enrolled	Out-migration
1081	Male	47	Rural	Central	Farmer	Relapse	Not enrolled	Belief of being cured
1082	Male	27	Rural	Southern	Farmer	Retreatment failure	Enrolled	
1083	Male	45	Rural	Southern	Farmer	Relapse	Not enrolled	Non-standard treatment
1084	Male	60	Rural	Southern	Farmer	New	Enrolled	
1085	Male	48	Rural	Southern	Farmer	New	Not enrolled	Belief of being cured
1086	Male	37	Rural	Southwestern	Farmer	Relapse	Enrolled	
1087	Male	18	Urban	Northern	City worker	Smear+ after 3 ms	Enrolled	
1088	Male	49	Rural	Southwestern	Farmer	Retreatment failure	Enrolled	
1089	Male	27	Urban	Central	Unemployed/unreported	Relapse	Enrolled	
1090	Male	29	Rural	Northern	City worker	Relapse	Enrolled	
1091	Male	37	Rural	Unknown	Farmer	Retreatment failure	Not enrolled	Unknown address
1092	Male	49	Rural	Southern	Farmer	New	Not enrolled	Death
1093	Male	29	Urban	Northern	City worker	Retreatment failure	Enrolled	
1094	Femal	20	Rural	Other province	City worker	Relapse	Enrolled	
1095	Femal	65	Urban	Northern	Retired/Student	New	Enrolled	

1096	Male	22	Urban	Central	City worker	New	Enrolled	
1097	Male	38	Urban	Central	City worker	New	Enrolled	
1098	Male	84	Rural	Central	Farmer	New	Not enrolled	Belief of being cured
1099	Male	45	Rural	Central	Farmer	Retreatment failure	Not enrolled	studies
1100	Femal	62	Rural	Unknown	Farmer	New	Not enrolled	Unknown address
1101	Male	37	Rural	Southern	Farmer	Relapse	Enrolled	
1102	Male	50	Urban	Southern	Unemployed/unreported	New	Not enrolled	Non-standard treatment
1103	Femal	41	Rural	Northern	Farmer	Retreatment failure	Enrolled	
1104	Male	31	Rural	Southern	Farmer	New	Not enrolled	Other severe disease
1105	Male	55	Urban	Northern	City worker	Relapse	Enrolled	
1106	Femal	33	Rural	Northern	Farmer	Retreatment failure	Enrolled	
1107	Femal	42	Urban	Northern	Unemployed/unreported	Retreatment failure	Enrolled	
1108	Male	56	Rural	Northern	Farmer	New	Not enrolled	Economic hardship
1109	Male	42	Rural	Southern	Farmer	Initial treatment failur	Not enrolled	Belief of being cured
1110	Male	46	Urban	Northern	Unemployed/unreported	Retreatment failure	Enrolled	
1111	Male	58	Rural	Central	Farmer	Retreatment failure	Enrolled	
1112	Male	52	Rural	Central	Farmer	Relapse	Not enrolled	Economic hardship
1113	Male	23	Rural	Northern	Farmer	Retreatment failure	Enrolled	
1114	Femal	84	Rural	Central	Farmer	Smear+ after 3 ms	Not enrolled	Other severe disease
1115	Femal	23	Rural	Southern	Farmer	Retreatment failure	Enrolled	
1116	Femal	42	Rural	Central	Farmer	Retreatment failure	Enrolled	
1117	Femal	49	Rural	Southern	Farmer	Initial treatment failur	Enrolled	
1118	Male	57	Rural	Northern	Farmer	Relapse	Enrolled	
1119	Male	50	Urban	Northern	City worker	Relapse	Enrolled	
1120	Male	47	Rural	Southwestern	City worker	Retreatment failure	Enrolled	
1121	Male	69	Rural	Northern	Farmer	New	Enrolled	
1122	Male	36	Urban	Southwestern	City worker	Retreatment failure	Enrolled	
1123	Femal	56	Urban	Southwestern	City worker	Retreatment failure	Enrolled	
1124	Male	44	Urban	Northern	City worker	Retreatment failure	Enrolled	
1125	Male	59	Urban	Southwestern	Unemployed/unreported	Initial treatment failur	Enrolled	
1126	Male	17	Urban	Southwestern	Retired/Student	New	Enrolled	
1127	Male	56	Rural	Central	Farmer	New	Not enrolled	Non-standard treatment
1128	Male	34	Rural	Central	Farmer	Initial treatment failur	Enrolled	
1129	Male	46	Rural	Western	Farmer	Relapse	Enrolled	
1130	Male	28	Rural	Southwestern	Farmer	New	Enrolled	
1131	Male	67	Rural	Southern	Farmer	Relapse	Not enrolled	Non-standard treatment
1132	Femal	22	Rural	Western	Farmer	Relapse	Enrolled	
1133	Femal	52	Urban	Southern	City worker	Retreatment failure	Enrolled	
1134	Male	49	Rural	Central	Farmer	Initial treatment failur	Not enrolled	Belief of being cured
1135	Male	47	Urban	Southwestern	Unemployed/unreported	New	Enrolled	
1136	Male	59	Rural	Southwestern	Farmer	Retreatment failure	Enrolled	
1137	Femal	27	Rural	Northern	Farmer	Retreatment failure	Enrolled	
1138	Femal	43	Rural	Southern	Farmer	Relapse	Enrolled	
1139	Femal	45	Rural	Southwestern	Farmer	Relapse	Enrolled	
1140	Male	70	Rural	Unknown	Farmer	Retreatment failure	Not enrolled	Unknown address
1141	Male	56	Rural	Central	Farmer	Relapse	Enrolled	
1142	Male	43	Rural	Northern	Farmer	Retreatment failure	Enrolled	
1143	Femal	35	Rural	Central	Farmer	Retreatment failure	Not enrolled	Out-migration
1144	Femal	40	Rural	Western	Farmer	Initial treatment failur	Enrolled	
1145	Femal	45	Urban	Northern	Unemployed/unreported	Retreatment failure	Enrolled	
1146	Male	57	Rural	Southwestern	Farmer	Relapse	Enrolled	
1147	Femal	37	Rural	Southwestern	Farmer	Retreatment failure	Enrolled	
1148	Male	46	Rural	Southwestern	Farmer	Retreatment failure	Enrolled	
1149	Male	51	Urban	Northern	Unemployed/unreported	Retreatment failure	Enrolled	
1150	Male	73	Rural	Southwestern	Farmer	Retreatment failure	Not enrolled	Death
1151	Male	12	Urban	Southwestern	Retired/Student	Retreatment failure	Enrolled	
1152	Male	40	Rural	Southwestern	Farmer	Relapse	Enrolled	
1153	Male	65	Urban	Northern	Retired/Student	Retreatment failure	Not enrolled	Other severe disease
1154	Male	63	Rural	Northern	Farmer	New	Not enrolled	Out-migration
1155	Femal	25	Rural	Other province	Farmer	Retreatment failure	Not enrolled	Migrants from other provinces
1156	Male	31	Rural	Western	Farmer	Relapse	Not enrolled	Out-migration

1157	Femal	80	Urban	Northern	Retired/Student	Initial treatment failur	Not enrolled	Other severe disease
1158	Male	76	Rural	Northern	Farmer	New	Enrolled	
1159	Femal	28	Urban	Southwestern	Unemployed/unreported	Retreatment failure	Enrolled	
1160	Femal	28	Rural	Central	Farmer	New	Enrolled	
1161	Male	53	Rural	Southwestern	Farmer	Retreatment failure	Not enrolled	Economic hardship
1162	Male	58	Rural	Central	Farmer	Retreatment failure	Enrolled	
1163	Male	66	Rural	Central	Retired/Student	Retreatment failure	Not enrolled	Economic hardship
1164	Male	54	Rural	Southern	Farmer	Retreatment failure	Enrolled	
1165	Male	61	Rural	Northern	Retired/Student	Relapse	Not enrolled	Economic hardship
1166	Male	41	Rural	Northern	Farmer	Relapse	Enrolled	
1167	Male	63	Rural	Northern	Retired/Student	New	Enrolled	
1168	Male	59	Rural	Southern	Farmer	New	Not enrolled	Economic hardship
1169	Male	59	Urban	Southwestern	Unemployed/unreported	Retreatment failure	Enrolled	
1170	Male	26	Urban	Southwestern	Unemployed/unreported	Initial treatment failur	Enrolled	
1171	Male	45	Rural	Central	Farmer	Retreatment failure	Enrolled	
1172	Male	64	Rural	Southwestern	Farmer	New	Not enrolled	Economic hardship
1173	Male	33	Urban	Southwestern	Unemployed/unreported	Relapse	Enrolled	
1174	Male	58	Urban	Southwestern	City worker	Relapse	Enrolled	
1175	Male	59	Rural	Southwestern	Farmer	Retreatment failure	Not enrolled	Death
1176	Male	31	Rural	Central	Farmer	New	Enrolled	
1177	Male	35	Urban	Southwestern	City worker	Relapse	Enrolled	
1178	Femal	23	Urban	Northern	City worker	Retreatment failure	Enrolled	
1179	Male	25	Rural	Southern	Farmer	Initial treatment failur	Enrolled	
1180	Male	77	Rural	Northern	Farmer	New	Not enrolled	studies
1181	Male	23	Rural	Western	Farmer	Retreatment failure	Not enrolled	Belief of being cured
1182	Male	43	Urban	Northern	City worker	Retreatment failure	Enrolled	
1183	Male	47	Rural	Central	Farmer	Relapse	Not enrolled	Belief of being cured
1184	Femal	46	Urban	Northern	Unemployed/unreported	Initial treatment failur	Enrolled	
1185	Male	28	Rural	Northern	Farmer	New	Enrolled	
1186	Femal	43	Urban	Southwestern	Unemployed/unreported	Retreatment failure	Enrolled	
1187	Male	23	Urban	Central	City worker	Retreatment failure	Enrolled	
1188	Femal	31	Urban	Northern	City worker	Relapse	Enrolled	
1189	Male	46	Urban	Northern	City worker	Retreatment failure	Enrolled	
1190	Male	33	Rural	Southwestern	Farmer	Relapse	Not enrolled	Other severe disease
1191	Male	37	Urban	Southwestern	City worker	Retreatment failure	Enrolled	
1192	Male	24	Urban	Northern	City worker	Retreatment failure	Enrolled	
1193	Male	73	Rural	Northern	Farmer	Retreatment failure	Enrolled	
1194	Femal	49	Rural	Southwestern	Farmer	Initial treatment failur	Not enrolled	Belief of being cured
1195	Male	61	Urban	Southern	Retired/Student	Retreatment failure	Enrolled	
1196	Femal	42	Rural	Southwestern	Farmer	Relapse	Enrolled	
1197	Male	42	Rural	Northern	Farmer	Retreatment failure	Enrolled	
1198	Male	76	Urban	Central	Retired/Student	Return or other	Enrolled	
1199	Femal	34	Urban	Northern	City worker	Retreatment failure	Enrolled	
1200	Male	83	Urban	Central	Retired/Student	New	Enrolled	
1201	Male	54	Rural	Northern	Farmer	Initial treatment failur	Not enrolled	Death
1202	Femal	48	Rural	Northern	Farmer	Retreatment failure	Enrolled	
1203	Male	51	Urban	Central	Unemployed/unreported	Retreatment failure	Not enrolled	studies
1204	Male	45	Rural	Central	Farmer	Relapse	Enrolled	
1205	Femal	47	Rural	Southwestern	Farmer	New	Enrolled	
1206	Male	55	Rural	Central	Farmer	Retreatment failure	Enrolled	
1207	Male	49	Rural	Central	Farmer	Relapse	Not enrolled	Economic hardship
1208	Femal	34	Urban	Central	Unemployed/unreported	Retreatment failure	Enrolled	
1209	Male	25	Rural	Southwestern	Farmer	Relapse	Enrolled	
1210	Male	54	Rural	Central	Farmer	Retreatment failure	Not enrolled	Belief of being cured
1211	Male	71	Rural	Central	Farmer	Relapse	Not enrolled	Other severe disease
1212	Male	70	Rural	Southwestern	Retired/Student	Retreatment failure	Not enrolled	Out-migration
1213	Femal	40	Rural	Southern	Farmer	Return or other	Not enrolled	Out-migration
1214	Femal	40	Rural	Unknown	Farmer	Retreatment failure	Not enrolled	Unknown address
1215	Male	46	Rural	Southern	Farmer	New	Not enrolled	Death
1216	Male	41	Rural	Central	Farmer	Retreatment failure	Not enrolled	Economic hardship
1217	Male	22	Urban	Central	Unemployed/unreported	Smear+ after 3 ms	Enrolled	

1218	Male	72	Rural	Central	Retired/Student	Retreatment failure	Enrolled	
1219	Male	26	Rural	Southwestern	Farmer	Initial treatment failur	Enrolled	
1220	Male	37	Urban	Northern	City worker	Relapse	Enrolled	
1221	Male	25	Urban	Northern	City worker	Initial treatment failur	Enrolled	
1222	Femal	33	Rural	Northern	Farmer	Relapse	Not enrolled	studies
1223	Male	27	Urban	Northern	Unemployed/unreported	Retreatment failure	Enrolled	
1224	Male	37	Rural	Western	Farmer	Relapse	Enrolled	
1225	Male	45	Rural	Northern	Farmer	Relapse	Enrolled	
1226	Male	35	Urban	Northern	Unemployed/unreported	Relapse	Enrolled	
1227	Male	49	Rural	Central	Farmer	Retreatment failure	Not enrolled	Belief of being cured
1228	Male	35	Urban	Northern	Unemployed/unreported	Retreatment failure	Enrolled	
1229	Male	71	Rural	Central	Farmer	New	Not enrolled	Economic hardship
1230	Femal	53	Rural	Central	Farmer	Relapse	Enrolled	
1231	Male	58	Rural	Northern	Farmer	New	Not enrolled	Non-standard treatment
1232	Male	67	Urban	Central	Retired/Student	Relapse	Not enrolled	Other severe disease
1233	Male	24	Rural	Southwestern	Farmer	Relapse	Not enrolled	Economic hardship
1234	Male	69	Rural	Northern	Farmer	Retreatment failure	Not enrolled	Death
1235	Male	22	Rural	Northern	Farmer	Retreatment failure	Enrolled	
1236	Male	39	Urban	Central	Unemployed/unreported	Initial treatment failur	Enrolled	
1237	Male	28	Rural	Northern	Farmer	Retreatment failure	Enrolled	
1238	Femal	40	Rural	Central	Farmer	New	Enrolled	
1239	Male	53	Rural	Southwestern	Farmer	New	Enrolled	
1240	Male	67	Urban	Northern	Retired/Student	Relapse	Enrolled	
1241	Male	69	Rural	Northern	Retired/Student	Retreatment failure	Enrolled	
1242	Male	52	Rural	Central	Farmer	Retreatment failure	Not enrolled	Economic hardship
1243	Male	53	Rural	Southwestern	Farmer	Relapse	Not enrolled	Non-standard treatment
1244	Femal	22	Rural	Northern	Farmer	Retreatment failure	Not enrolled	Non-standard treatment
1245	Femal	27	Rural	Southwestern	Farmer	Retreatment failure	Enrolled	
1246	Femal	50	Rural	Central	Farmer	Relapse	Enrolled	
1247	Male	75	Rural	Southwestern	Retired/Student	Retreatment failure	Not enrolled	Death
1248	Male	35	Rural	Central	Farmer	Retreatment failure	Enrolled	
1249	Male	44	Rural	Southwestern	Farmer	Relapse	Enrolled	
1250	Male	47	Rural	Central	Farmer	Retreatment failure	Enrolled	
1251	Male	54	Rural	Northern	Farmer	Retreatment failure	Not enrolled	Economic hardship
1252	Femal	54	Rural	Southwestern	Farmer	Retreatment failure	Not enrolled	Belief of being cured
1253	Male	46	Urban	Northern	Farmer	Relapse	Not enrolled	Economic hardship
1254	Male	20	Rural	Southern	Farmer	New	Enrolled	
1255	Male	31	Rural	Southwestern	Farmer	Relapse	Not enrolled	Economic hardship
1256	Male	58	Rural	Northern	Farmer	New	Enrolled	
1257	Male	40	Rural	Southwestern	Farmer	Relapse	Not enrolled	Belief of being cured
1258	Male	43	Rural	Southwestern	Farmer	Initial treatment failur	Enrolled	
1259	Male	21	Rural	Southwestern	Farmer	Retreatment failure	Enrolled	
1260	Male	45	Rural	Southern	Farmer	Retreatment failure	Not enrolled	Economic hardship
1261	Male	31	Rural	Southern	Farmer	Retreatment failure	Enrolled	
1262	Male	42	Rural	Southern	Farmer	Relapse	Enrolled	
1263	Male	44	Rural	Northern	Farmer	Retreatment failure	Enrolled	
1264	Femal	38	Rural	Central	Farmer	New	Enrolled	
1265	Male	70	Rural	Northern	Retired/Student	New	Not enrolled	Other severe disease
1266	Male	21	Rural	Central	Farmer	Relapse	Enrolled	
1267	Male	49	Urban	Northern	Unemployed/unreported	New	Enrolled	
1268	Male	60	Rural	Southern	Farmer	Relapse	Not enrolled	Non-standard treatment
1269	Male	49	Rural	Northern	Farmer	Relapse	Enrolled	
1270	Femal	42	Rural	Western	Farmer	Retreatment failure	Enrolled	
1271	Femal	45	Rural	Southwestern	Farmer	Initial treatment failur	Enrolled	
1272	Male	41	Rural	Southern	Farmer	Retreatment failure	Enrolled	
1273	Femal	35	Urban	Southwestern	City worker	Initial treatment failur	Not enrolled	Belief of being cured
1274	Male	64	Urban	Northern	Retired/Student	New	Enrolled	
1275	Male	48	Rural	Southwestern	Farmer	Relapse	Not enrolled	Economic hardship
1276	Male	53	Rural	Southern	Farmer	Retreatment failure	Enrolled	
1277	Femal	37	Rural	Southwestern	Farmer	Retreatment failure	Not enrolled	Out-migration
1278	Male	41	Rural	Central	Farmer	New	Enrolled	

1279	Male	40	Rural	Western	Farmer	Retreatment failure	Not enrolled	Belief of being cured
1280	Male	44	Rural	Other province	Farmer	Retreatment failure	Enrolled	
1281	Male	62	Rural	Southern	Retired/Student	Retreatment failure	Not enrolled	Economic hardship
1282	Male	53	Rural	Northern	Farmer	New	Enrolled	
1283	Male	56	Rural	Southwestern	Farmer	Retreatment failure	Not enrolled	Death
1284	Male	46	Rural	Central	Farmer	Relapse	Enrolled	
1285	Male	41	Rural	Central	Farmer	Retreatment failure	Enrolled	
1286	Male	50	Rural	Southwestern	Farmer	Retreatment failure	Enrolled	
1287	Femal	24	Rural	Southwestern	Farmer	Relapse	Enrolled	
1288	Male	50	Rural	Central	Farmer	Initial treatment failur	Not enrolled	Belief of being cured
1289	Male	35	Rural	Central	Farmer	New	Not enrolled	Belief of being cured
1290	Male	42	Urban	Northern	Unemployed/unreported	Retreatment failure	Enrolled	
1291	Male	61	Urban	Western	Retired/Student	Retreatment failure	Enrolled	
1292	Male	59	Rural	Southwestern	Farmer	New	Not enrolled	Economic hardship
1293	Male	55	Rural	Southern	Farmer	Relapse	Enrolled	
1294	Male	45	Rural	Southern	Farmer	Retreatment failure	Not enrolled	Concern about work and studies
1295	Male	57	Urban	Southwestern	City worker	Retreatment failure	Enrolled	
1296	Femal	37	Rural	Southwestern	Farmer	Relapse	Enrolled	
1297	Male	53	Rural	Southwestern	Farmer	Relapse	Enrolled	
1298	Male	49	Rural	Southern	Farmer	Retreatment failure	Enrolled	
1299	Male	20	Urban	Northern	Retired/Student	Retreatment failure	Enrolled	
1300	Male	62	Rural	Central	Retired/Student	New	Enrolled	
1301	Male	16	Rural	Northern	Retired/Student	Relapse	Enrolled	
1302	Male	50	Rural	Southwestern	Farmer	Relapse	Enrolled	
1303	Male	60	Rural	Northern	Farmer	Retreatment failure	Enrolled	
1304	Femal	41	Rural	Southern	Farmer	Relapse	Enrolled	
1305	Male	21	Rural	Southwestern	Farmer	New	Enrolled	
1306	Male	42	Rural	Southwestern	Farmer	Retreatment failure	Not enrolled	Economic hardship
1307	Male	37	Rural	Southern	Farmer	Retreatment failure	Enrolled	
1308	Male	38	Rural	Northern	Farmer	New	Not enrolled	Belief of being cured
1309	Femal	47	Rural	Western	Farmer	Retreatment failure	Enrolled	
1310	Male	26	Rural	Southwestern	Farmer	New	Enrolled	
1311	Femal	67	Rural	Southwestern	Retired/Student	Retreatment failure	Enrolled	
1312	Femal	67	Urban	Central	City worker	Retreatment failure	Enrolled	
1313	Male	31	Rural	Northern	Farmer	Retreatment failure	Enrolled	
1314	Male	64	Rural	Northern	Retired/Student	Retreatment failure	Not enrolled	Death
1315	Male	43	Urban	Northern	Unemployed/unreported	Relapse	Not enrolled	studies
1316	Femal	29	Rural	Southern	Farmer	Initial treatment failur	Enrolled	
1317	Male	58	Rural	Southern	Farmer	Relapse	Enrolled	
1318	Male	40	Urban	Western	City worker	Retreatment failure	Enrolled	
1319	Femal	23	Rural	Western	Farmer	Initial treatment failur	Enrolled	
1320	Femal	31	Rural	Southern	Farmer	Retreatment failure	Enrolled	
1321	Male	41	Rural	Northern	Farmer	Retreatment failure	Enrolled	
1322	Male	57	Rural	Northern	Farmer	Retreatment failure	Not enrolled	Economic hardship
1323	Male	66	Rural	Central	Farmer	Retreatment failure	Not enrolled	Economic hardship
1324	Male	44	Rural	Northern	Farmer	Relapse	Not enrolled	Economic hardship
1325	Male	23	Rural	Southern	Farmer	New	Not enrolled	Belief of being cured
1326	Male	56	Rural	Northern	Farmer	Retreatment failure	Enrolled	
1327	Male	25	Rural	Western	Farmer	Retreatment failure	Enrolled	
1328	Male	63	Rural	Northern	Farmer	Relapse	Not enrolled	Death
1329	Male	32	Rural	Central	Farmer	Relapse	Not enrolled	Economic hardship
1330	Femal	52	Rural	Southwestern	Farmer	Retreatment failure	Enrolled	
1331	Male	35	Rural	Southwestern	Farmer	Initial treatment failur	Not enrolled	Economic hardship
1332	Male	58	Rural	Northern	Farmer	Initial treatment failur	Enrolled	
1333	Male	32	Rural	Western	Farmer	Retreatment failure	Enrolled	
1334	Male	23	Urban	Central	Unemployed/unreported	Retreatment failure	Enrolled	
1335	Femal	34	Rural	Southern	Farmer	Retreatment failure	Enrolled	
1336	Femal	68	Rural	Northern	Farmer	Retreatment failure	Not enrolled	Economic hardship
1337	Femal	42	Rural	Other province	Farmer	New	Enrolled	
1338	Femal	23	Rural	Southwestern	Farmer	New	Enrolled	
1339	Femal	23	Rural	Southwestern	Farmer	New	Enrolled	

1340	Femal	26	Rural	Other province	Farmer	Relapse	Not enrolled	Migrants from other provinces
1341	Femal	21	Rural	Western	Farmer	Retreatment failure	Enrolled	
1342	Male	69	Urban	Western	City worker	Initial treatment failur	Enrolled	
1343	Male	47	Rural	Southwestern	Farmer	New	Enrolled	
1344	Male	59	Rural	Northern	Farmer	New	Enrolled	
1345	Male	48	Rural	Southern	Farmer	Retreatment failure	Enrolled	
1346	Femal	58	Rural	Northern	Farmer	Retreatment failure	Enrolled	
1347	Male	26	Rural	Central	Farmer	Retreatment failure	Enrolled	
1348	Femal	26	Rural	Southern	Farmer	Relapse	Enrolled	
1349	Male	63	Rural	Northern	Farmer	Relapse	Not enrolled	studies
1350	Male	44	Rural	Southwestern	Farmer	Retreatment failure	Enrolled	
1351	Male	47	Rural	Central	Farmer	Relapse	Not enrolled	Belief of being cured
1352	Male	56	Rural	Central	Farmer	Relapse	Not enrolled	Belief of being cured
1353	Male	33	Rural	Western	Farmer	New	Enrolled	
1354	Male	48	Rural	Southern	Farmer	Relapse	Enrolled	
1355	Male	67	Rural	Southern	Farmer	Initial treatment failur	Enrolled	
1356	Male	58	Urban	Southern	Unemployed/unreported	Initial treatment failur	Enrolled	
1357	Male	76	Rural	Southwestern	Farmer	Relapse	Not enrolled	Economic hardship
1358	Femal	44	Rural	Central	Farmer	New	Not enrolled	Belief of being cured
1359	Femal	37	Rural	Unknown	Farmer	Retreatment failure	Not enrolled	Unknown address
1360	Femal	25	Rural	Southern	Farmer	Retreatment failure	Enrolled	
1361	Femal	29	Rural	Southern	Farmer	Retreatment failure	Enrolled	
1362	Femal	19	Rural	Southern	Retired/Student	Retreatment failure	Enrolled	
1363	Male	45	Rural	Central	Farmer	Relapse	Enrolled	
1364	Femal	19	Rural	Southern	Retired/Student	Retreatment failure	Enrolled	
1365	Male	58	Rural	Southern	Farmer	Retreatment failure	Enrolled	
1366	Femal	51	Rural	Southern	Farmer	Relapse	Enrolled	
1367	Male	51	Rural	Southern	Farmer	Retreatment failure	Not enrolled	Death
1368	Male	58	Rural	Southern	Farmer	Relapse	Not enrolled	Non-standard treatment
1369	Male	49	Rural	Southern	Farmer	Relapse	Enrolled	
1370	Male	48	Rural	Southern	Farmer	Retreatment failure	Enrolled	
1371	Male	62	Rural	Central	Farmer	Relapse	Enrolled	
1372	Male	21	Rural	Central	Farmer	Retreatment failure	Enrolled	
1373	Male	23	Rural	Central	Farmer	New	Not enrolled	Economic hardship
1374	Male	69	Rural	Central	Farmer	Retreatment failure	Not enrolled	Belief of being cured
1375	Male	46	Rural	Northern	Farmer	Relapse	Enrolled	
1376	Femal	23	Urban	Southwestern	Unemployed/unreported	Relapse	Enrolled	
1377	Femal	24	Rural	Southwestern	Farmer	Retreatment failure	Not enrolled	Death
1378	Male	56	Urban	Central	City worker	Relapse	Not enrolled	Belief of being cured
1379	Male	66	Rural	Southern	Farmer	New	Not enrolled	Belief of being cured
1380	Male	31	Rural	Northern	Farmer	Retreatment failure	Not enrolled	Other severe disease
1381	Male	53	Rural	Northern	Farmer	New	Not enrolled	Death
1382	Femal	22	Urban	Northern	City worker	Retreatment failure	Enrolled	
1383	Femal	27	Urban	Northern	City worker	Initial treatment failur	Enrolled	
1384	Male	22	Rural	Southern	Farmer	Retreatment failure	Enrolled	
1385	Male	68	Rural	Southwestern	Retired/Student	Smear+ after 3 ms	Not enrolled	Death
1386	Femal	32	Rural	Southern	Farmer	Relapse	Enrolled	
1387	Femal	64	Rural	Northern	Farmer	Relapse	Not enrolled	Belief of being cured
1388	Male	65	Rural	Northern	Farmer	Relapse	Enrolled	
1389	Male	45	Rural	Western	Farmer	Initial treatment failur	Enrolled	
1390	Male	34	Rural	Southern	Farmer	Smear+ after 3 ms	Not enrolled	Out-migration
1391	Male	64	Urban	Northern	Retired/Student	Initial treatment failur	Not enrolled	Death
1392	Femal	36	Rural	Northern	Farmer	New	Not enrolled	Belief of being cured
1393	Male	52	Rural	Northern	Farmer	Relapse	Enrolled	
1394	Male	15	Rural	Northern	Unemployed/unreported	Retreatment failure	Not enrolled	studies
1395	Femal	71	Rural	Northern	Farmer	Relapse	Not enrolled	Non-standard treatment
1396	Male	69	Rural	Central	Farmer	Relapse	Not enrolled	Economic hardship
1397	Male	38	Rural	Southwestern	Farmer	Relapse	Enrolled	
1398	Male	23	Rural	Central	Farmer	Retreatment failure	Not enrolled	Economic hardship
1399	Male	21	Urban	Southwestern	City worker	Relapse	Enrolled	
1400	Male	15	Rural	Other province	Retired/Student	Initial treatment failur	Enrolled	

1401	Femal	48	Rural	Southwestern	Farmer	Retreatment failure	Enrolled	
1402	Male	55	Rural	Northern	Farmer	Relapse	Not enrolled	Economic hardship
1403	Femal	52	Rural	Central	Farmer	Initial treatment failur	Enrolled	
1404	Male	38	Urban	Southern	Unemployed/unreported	Retreatment failure	Enrolled	
1405	Male	29	Rural	Southern	Farmer	Retreatment failure	Enrolled	
1406	Femal	49	Rural	Southwestern	Farmer	New	Enrolled	
1407	Male	61	Rural	Northern	Farmer	Initial treatment failur	Not enrolled	Non-standard treatment
1408	Male	37	Rural	Southern	Farmer	Relapse	Enrolled	
1409	Femal	21	Rural	Unknown	Farmer	Relapse	Not enrolled	Unknown address
1410	Femal	23	Rural	Central	Farmer	Relapse	Enrolled	
1411	Male	46	Rural	Southwestern	Farmer	Retreatment failure	Not enrolled	Out-migration
1412	Femal	25	Rural	Southwestern	Farmer	Retreatment failure	Enrolled	
1413	Male	46	Rural	Southwestern	Farmer	Retreatment failure	Not enrolled	Economic hardship
1414	Male	51	Rural	Central	Farmer	Relapse	Not enrolled	Belief of being cured
1415	Male	55	Rural	Central	Farmer	New	Not enrolled	Economic hardship
1416	Male	43	Rural	Southwestern	Farmer	Retreatment failure	Not enrolled	Out-migration
1417	Femal	41	Rural	Southwestern	Farmer	Retreatment failure	Enrolled	
1418	Male	44	Rural	Northern	Farmer	Relapse	Enrolled	
1419	Femal	33	Rural	Northern	Farmer	Relapse	Enrolled	
1420	Male	48	Rural	Central	Farmer	Initial treatment failur	Enrolled	
1421	Male	31	Rural	Western	Farmer	Retreatment failure	Enrolled	
1422	Male	43	Rural	Southwestern	Farmer	Retreatment failure	Enrolled	
1423	Male	44	Rural	Central	Farmer	Relapse	Enrolled	
1424	Male	42	Rural	Central	Farmer	Retreatment failure	Enrolled	
1425	Femal	41	Rural	Unknown	Farmer	Relapse	Not enrolled	Unknown address
